# Crystal Face‐Dependent Behavior of Single‐Atom Pt: Construct of SA‐FLP Dual Active Sites for Efficient NO_2_ Detection

**DOI:** 10.1002/advs.202402038

**Published:** 2024-05-29

**Authors:** Yucheng Ou, Bing Wang, Nana Xu, Quzhi Song, Tao Liu, Hui Xu, Fuwen Wang, Yingde Wang

**Affiliations:** ^1^ Science and Technology on Advanced Ceramic Fiber and Composites Laboratory College of Aerospace Science and Engineering National University of Defense Technology Changsha 410073 China

**Keywords:** CeO_2_, gas sensor, FLP, NO_2_ detection, Pt single atom

## Abstract

The strong potential of platinum single atom (Pt_SA_) in gas sensor technology is primarily attributed to its high atomic economy. Nevertheless, it is imperative to conduct further exploration to understand the impact of Pt_SA_ on the active sites. In this study, the evolution of Pt_SA_ on (100)CeO_2_ and (111)CeO_2_ is examined, revealing notable disparities in the position and activity of surface Pt_SA_ on different crystal planes. The Pt_SA_ in (100)CeO_2_ surface can enhance the stability of Ce^3+^ and construct a frustrated Lewis pair (FLP) to form a double active site by combining the steric hindrance effect of oxygen vacancies, which increases the response value from 1.8 to 27 and reduce the response‐recovery time from 140–192 s to 25–26 s toward five ppm NO_2_ at room temperature. Conversely, Pt_SA_ tends to bind to terminal oxygen on the surface of (111)CeO_2_ and become an independent reaction site. The response value of Pt_SA_‐(111)CeO_2_ surface only increased from 1.6 to 3.8. This research underscores the correlation between single atoms and crystal plane effects, laying the groundwork for designing and synthesizing ultra‐stable and efficient gas sensors.

## Graphical abstract

The Pt_SA_ in (100)CeO_2_ surface enhances the stability of Ce^3+^ and constructs frustrated Lewis pair to form a double active site by combining the steric hindrance effect of oxygen vacancies, which increases the response value from 1.8 to 27 and reduces response‐recovery time from 140–192 s to 25–26 s toward five ppm NO_2_ at room temperature.

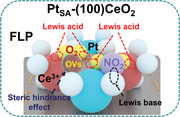

## 1 Introduction

The development of gas sensors that can quickly and stably detect NO2 in the ppb range is essential for real‐time monitoring of air quality in chemical plants.^[^ ^1^, ^2^, ^3^ ^]^ Chemiresistive sensors have been applied to detect NO_2_ because of their high efficiency and sensitivity.^[^ ^4^, ^5^, ^6^ ^]^ Unfortunately, the slower response‐recovery time and baseline resistance drift of chemiresistive sensors remain essential parameters that need to be improved for practical applications.^[^ ^7^, ^8^, ^9^ ^]^ The gas sensing mechanism of chemiresistive sensors is based on the surface reaction between target gas and activity oxygen on the surface of the sensitive layer.^[^ ^10^, ^11^, ^12^ ^]^ Therefore, achieving simultaneous optimization of dynamics and thermodynamics to enhance the surface reaction efficiency and reaction activity of chemiresistive sensors for the detection of NO_2_ is essential for developing the next‐generation sensors for NO_2_ detection with high efficiency and superior stability at room temperature for practical applications.

Due to the fully surface‐exposed and atomically dispersed active sites, single‐atom catalysts (SAs) have received extensive attention in gas sensors.^[^ ^13^, ^14^, ^15^ ^]^ The introduction of SAs in sensitive layers can promote the adsorption‐dissociation of oxygen molecules and target gas, reducing the activation energy of surface reaction and enhancing the sensitive layer's electron transfer ability.^[^ ^16^, ^17^, ^18^, ^19^ ^]^ The atomic dispersion of Pt in CeO_2_ as a support can be achieved by utilizing the strong metal‐support interactions (SMSIs) between Pt and CeO_2_ through oxidizing treatment at 800 °C.^[^ ^20^, ^21^, ^22^ ^]^ Additionally, the exact structure and reactivity activity of Pt_SA_, particularly the structure‐function relationship of SMSIs between CeO_2_ crystal face and Pt_SA_, is closely related to gas‐sensing performance. Previous studies have confirmed that the formation of Pt_SA_‐CeO_2_ composites by Pt_SA_ at different crystal facets of CeO_2_ exhibits significant differences in catalytic properties under the change of reaction atmosphere or operating temperature.^[^ ^23^, ^24^, ^25^, ^26^, ^27^ ^]^ The coordination difference of Pt_SA_ on different crystal faces of CeO_2_ leads to the difference in the influence of Pt_SA_ on the surface structure, which can generate a new and more active state. Therefore, revealing the correlation between single atoms and crystal plane effects is essential to the design and synthesis of ultra‐stable and efficient gas sensors. Recently, constructing frustrated Lewis pairs (FLPs) by combining the steric hindrance effect of oxygen vacancies has been an efficient method for the activity of small molecules.^[^ ^28^, ^29^, ^30^ ^]^ For example, the formation of FLPs in In_2_O_3‐x_ via combining coordinately unsaturated indium and a Lewis primary surface hydroxide site exhibits excellent catalytic performance toward CO_2_ reduction.^[^ ^31^ ^]^ (100)CeO_2_ has many surface oxygen vacancies due to the strong mutual repulsion between the oxygen ions on the surface, which act as the active site in the catalytic reaction process. The abundance of oxygen vacancies on the surface of (100)CeO_2_ promotes the generation of Ce^3+^, which is essential to generate the new Lewis acidic center. However, the presence of oxygen molecules and NO_2_ during the detection process leads to a decrease in the concentration of Ce^3+^ and oxygen vacancies, which destroys the stability of FLP in (100)CeO_2_. Consequently, leveraging the SMSIs between Pt_SA_ and (100)CeO_2_ to enhance the stability of Ce^3+^ and establish a dual‐active site constitutes an effective strategy for developing an efficient and stable NO_2_ detection chemoresistive sensor at room temperature.

Here, Pt_SA_ is loaded on the surfaces of (100)CeO_2_ and (111)CeO_2_ via 800 °C high‐temperature heat treatment, and the significant differences in the position and activity of Pt_SA_ on (100)CeO_2_ and (111)CeO_2_ are revealed, as well as the effect of Pt_SA_ on the surface structure of the supports. The introduction of Pt_SA_ in (100)CeO_2_ can improve the stability of Ce^3+^ to construct a stable FLP and a highly active single atom on the surface of (100)CeO_2_ to form a double active site, which increases the response value of (100)CeO_2_ from 1.8 to 27, and a reduction in the response‐recovery time from 140–192 s to 25–26 s toward 5 ppm NO_2_ at room temperature. Conversely, Pt_SA_ tends to bind to terminal oxygen on the surface of (111)CeO_2_ and become an independent reaction site. The response value of Pt_SA_‐(111)CeO_2_ surface only increased from 1.6 to 3.8. This study leverages the SMSI phenomenon between a single atom and the crystal plane to establish a dual‐active site, thereby enabling the efficient and stable detection of NO_2_.

## 2 Results and Discussion

### 2.1 Structural Characterization of Various Sample

**Figure 1:**
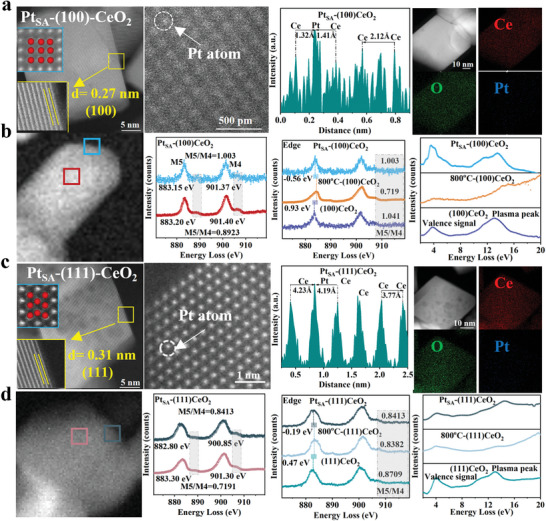
a) HAADF images of Pt_SA_‐(100)CeO_2_; HAADF images of Pt_SA_‐(100)CeO_2_ from low‐magnification; The atom distance of Pt_SA_‐(100)CeO_2_; EDS mapping of Pt_SA_‐(100)CeO_2_. b) HAADF images of Pt_SA_‐(100)CeO_2_ and corresponding valence electron energy loss spectra. c) HAADF images of Pt_SA_‐(111)CeO_2_; HAADF images of Pt_SA_‐(111)CeO_2_ from low‐magnification; The atom distance of Pt_SA_‐(111)CeO_2_; EDS mapping of Pt_SA_‐(111)CeO_2_. d) HAADF images of Pt_SA_‐(111)CeO_2_ and corresponding valence electron energy loss spectra.

The synthesis diagrams of Pt_SA_‐(111)CeO_2_ and Pt_SA_‐(100)CeO_2_ are shown in Figure S1 (Supporting Information). In order to understand the essential properties of single atomic and deepen the comprehension of the structure‐performance relationship, the Pt_SA_‐(100)CeO_2_ and Pt_SA_‐(111)CeO_2_ are comprehensively characterized. The X‐ray diffraction patterns analysis confirms that the prepared samples have the same cubic phase structure and the sample's crystallinity is improved after high‐temperature treatment. In particular, no characteristic peaks of Pt nanoparticles are detected (Figure S2, Supporting Information). Atomic resolution high angle annular dark field scanning transmission electron microscopy (AC HAADF‐STEM) provided microstructure visualization at the atom level, confirming the presence of Pt state on the surface of (100)CeO_2_ is a homogeneous atomic state. (**Figure** **1**a; Figure S3, Supporting Information). Additionally, the HAADF images confirm that the distance of Pt‐Ce is ≈1.4 Å, which is smaller than that of Ce‐Ce. The EDS element mapping images confirm the presence of Ce, O, and Pt elements on the surface of Pt_SA_‐(100)CeO_2_. The influence of Pt_SA_ toward (100)CeO_2_ surface is explored via electron energy loss spectrum (EELS). We selected the edge area of the particle for comparison with the center area of the particle (Figure 1b). The Zero‐loss peak of (100)CeO_2_, 800 °C‐(100)CeO_2_ and Pt_SA_‐(100)CeO_2_ are shown in Figure S4 (Supporting Information). The intensity and position of Ce‐M5 and Ce‐M4 edges are associated with Ce^4+^ and Ce^3+^. The peak positions of M5 and M4 for the edge area are lower than that of the center area, and the intensity ratios between M5 and M4 of the edge area are higher than that of the center area, which confirms the concentration of Ce^3+^ at the edge area is bigger than that of the center area. The peak intensity at 886.5 eV and 906.5 eV also confirms that Ce^4+^ concentrations are higher in the central region than in the peripheral region. The EELS analysis of (100)CeO_2_ and 800 °C‐(100)CeO_2_ are shown in Figures S5 and S6 (Supporting Information), the concentration of Ce^3+^ at the edge area is also bigger than that of the center area. Notably, compared with the peak position and the intensity ratios between M5 and M4 of (100)CeO_2_, the peak position of 800 °C‐(100)CeO_2_ shift to higher energy loss and the intensity ratios between M5 and M4 is 0.719, indicating the concentration of Ce^3+^ is significantly decrease due to high‐temperature treatment. However, the peak position of Pt_SA_‐(100)CeO_2_ shifts to lower energy loss compared with 800 °C‐(100)CeO_2_, and the intensity ratios between M5 and M4 of Pt_SA_‐(100)CeO_2_ are higher than that of 800 °C‐(100)CeO_2_. Therefore, the unique electron structure and chemical coordination environment of Pt_SA_ on the (100)CeO_2_ surface can maintain the surface Ce^3+^ activity site concentration. Similarly, the plasma signal intensity of Pt_SA_‐(100)CeO_2_ is higher than that of 800 °C‐(100)CeO_2_, indicating that the higher excitation electron concentration of Pt_SA_‐(100)CeO_2_ is greater than that of 800 °C‐(100)CeO_2_.

The HAADF image of Pt_SA_‐(111)CeO_2_ confirms the presence of the Pt state on the surface of (111)CeO_2_, which is also an atomic state (Figure 1c; Figure S7, Supporting Information). The distance of Pt‐Ce is ≈4.2 Å, which is more significant than that of Ce‐Ce. Therefore, the chemical coordination environment of Pt_SA_ on the (111)CeO_2_ surface differs from that on the (100)CeO_2_ surface. The EDS element mapping images also confirm the presence of Ce, O, and Pt elements on the surface of Pt_SA_‐(111)CeO_2_. We also selected the edge area and center area of (111)CeO_2_ to explore the influence of Pt_SA_ toward surface electron structure (Figure 1d). The Zero‐loss peak of (111)CeO_2_, 800 °C‐(111)CeO_2_ and Pt_SA_‐(111)CeO_2_ are shown in Figure S8 (Supporting Information). Comparing the peak position and intensity ratio of M5 and M4 at the edge region with that of the central region, the concentration of Ce^3+^ at the edge area is also more significant than that of the center area. The EELS analysis of (111)CeO_2_ and 800 °C‐(111)CeO_2_ also confirms that the concentration of Ce^3+^ at the edge area is more significant than that of the center area. Compared with the peak position and intensity ratios of M5 and M4 at (111)CeO_2_ and 800 °C‐(111)CeO_2_, the effect of high‐temperature treatment on Ce^3+^ concentration of (111)CeO_2_ is much less than that on (111)CeO_2_ (Figures S9 and S10, Supporting Information). Additionally, the peak position and intensity ratios of M5 and M4 at Pt_SA_‐(111)CeO_2_ are close to that of M5 and M4 in 800 °C‐(111)CeO_2_, indicating that the introduction of Pt_SA_ on the surface (111)CeO_2_ is not significantly changing the surface electron structure. Similarly, compared with the plasma signal intensity of 800 °C‐(100)CeO_2_, the introduction of Pt_SA_ can generate a higher excitation electron. Hence, according to the Pt‐Ce atom distance and the change of surface electron structure for (100)CeO_2_ and (111)CeO_2_, the surface chemical coordination environment of Pt_SA_ on the (100)CeO_2_ is different from that on the (111)CeO_2_ face.

**Figure 2:**
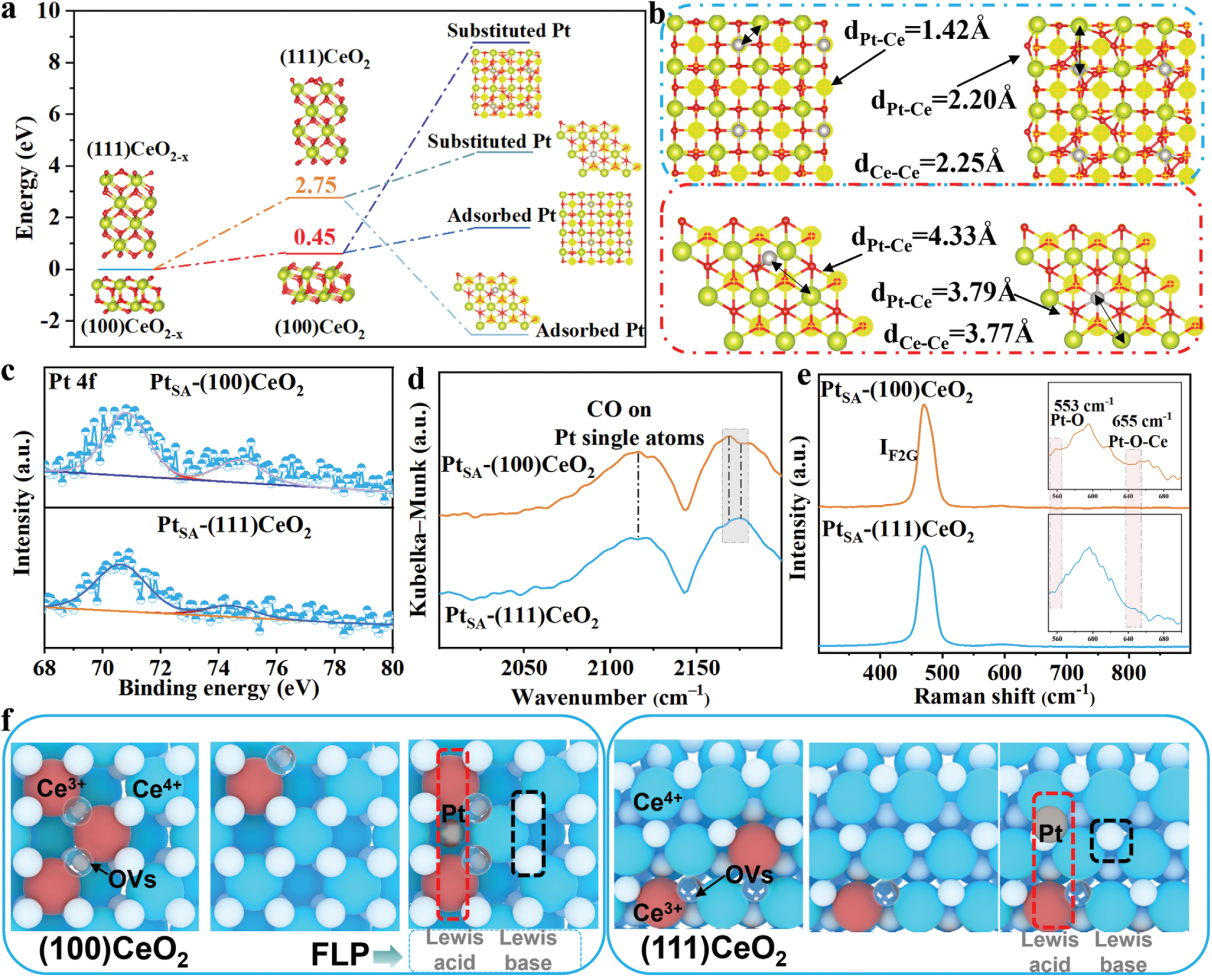
a) The formation energy of Pt_SA_‐(100)CeO_2_ and Pt_SA_‐(111)CeO_2_ calculated by DFT. b) The bong length of Pt‐Ce and Ce‐Ce toward Pt_SA_‐(100)CeO_2_ and Pt_SA_‐(111)CeO_2_. c) Pt 4f high‐resolution XPS spectra of Pt_SA_‐(100)CeO_2_ and Pt_SA_‐(111)CeO_2_. d) In situ FTIR spectra during CO adsorbed on the surface of Pt_SA_‐(100)CeO_2_ and Pt_SA_‐(111)CeO_2_. e) Raman spectra of Pt_SA_‐(100)CeO_2_ and Pt_SA_‐(111)CeO_2_. f) The structure diagram of Pt_SA_‐(100)CeO_2_ and Pt_SA_‐(111)CeO_2_.

The evolution process of Pt_SA_ on (111)CeO_2_ and (100)CeO_2_ surface during high‐temperature treatment under moving air atmosphere is investigated via density functional theory (DFT). The surface energies of (100)CeO2 with various layers are shown in Figure S11 (Supporting Information). When the surface energy increases, the surface becomes more unstable, which can easily lead to reconstruction. A relatively low surface energy suggests a more stable catalyst surface will often result in less activity. Therefore, the surface energy value of three layers is chosen, which can ensure sufficient active sites and is not easy to reconstruct. As shown in **Figure** **2**a, the surface adsorption O_2_ can occupy (100)CeO_2_ and (111)CeO_2_ surface oxygen vacancies site, and the reaction barrier of (100)CeO_2_ and (111)CeO_2_ are 0.45 and 2.75 eV implies that the surface structure stability of (100)CeO_2_ is weaker than that of (111)CeO_2_. Then, the chemical coordination environment of Pt_SA_ on (100)CeO_2_ and (111)CeO_2_ is further explored for various locations and binding geometries. We considered whether Pt_SA_ can adsorb on the surface or Pt can substitute the Ce atom on the surface. The reaction energy barrier of Pt_SA_ occupying the four‐fold hollow sites on (100)CeO_2_ surface is 0.90 eV, while the reaction energy barrier of Pt substituting Ce atom is 8.73 eV. The reaction energy barrier of Pt_SA_ adsorbing on (111)CeO_2_ surface is −4.87 eV, and the reaction energy barrier of Pt substituting Ce atom is 1.52 eV.

According to the analysis of AC‐HAADF STEM images and theoretical model for Pt‐Ce and Ce‐Ce atom distance (Figure 2b), the  Pt tends to occupy the four‐fold hollow sites on (100)CeO_2_ surface under oxidizing conditions at elevated temperatures. In contrast, the Pt_SA_ tends to adsorb on (111)CeO_2_ surface. The Pt 4f high‐resolution X‐ray photoelectron spectroscopy (XPS) spectra confirm that the valance state of Pt_SA_ is Pt^2+^ on the (100)CeO_2_ and (111)CeO_2_ surfaces (Figure 2c).^[^ ^32^ ^]^ Ce 3d and O 1s high‐resolution XPS spectra of various samples are shown in Figures S12 and S13 (Supporting Information). The Ce^3+^ and oxygen vacancies of (100)CeO_2_ are significantly decreased due to high‐temperature treatment. At the same time, the introduction of Pt_SA_ can change the surface electron structure to alleviate the decreasing trend of Ce^3+^ and oxygen vacancy concentration.  The low concentration of Ce^3+^ and oxygen vacancies on (111)CeO_2_ surface leads to the structure stability of (111)CeO_2_ being stronger than that of (111)CeO_2_, which decreases the influence of high temperature. The difference between Pt_SA_‐(100)CeO_2_ and Pt_SA_‐(111)CeO_2_ surface structure is further explored via diffuse‐reflectance infrared Fourier transform spectra with CO as a probe molecule (Figure 2d). The peaks at 2116 cm^−1^ corresponding to CO linear absorption peak, which confirmed that the presence state of Pt on the Pt_SA_‐(100)CeO_2_ and Pt_SA_‐(111)CeO_2_ are single atom. Precious studies have confirmed that the peaks at 2170 cm^−1^ are linked with CO adsorption at Ce^3+^ defect sites. The red‐shifted peak of Pt_SA_‐(100)CeO_2_ at 2168 cm^−1^ can be attributed to the Pt_SA_ occupying the fourfold hollow sites, which change the surface chemical environment of (100)CeO_2_. Meanwhile, the blue‐shifted peak of Pt_SA_‐(111)CeO_2_ at 2175 cm^−1^ can be attributed to CO adsorbed on the Ce atom near Pt_SA_. The Raman spectra of Pt_SA_‐(100)CeO_2_ and Pt_SA_‐(111)CeO_2_ show two peaks at 553 and 655 cm^−1^, which are assigned to Pt‐O and Pt‐O‐Ce, respectively (Figure 2e). Based on the above analysis, the microstructure schematic diagram of Pt_SA_‐(100)CeO_2_ and Pt_SA_‐(111)CeO_2_ are shown in Figure 2f. The introduction of Pt_SA_ on the four‐fold hollow sites can maintain the electron structure of Ce^3+^ (Lewis acid sites) and provide a new adsorption‐reaction site. They are combined with the Steric hindrance effect of oxygen vacancies to form a Pt_SA_‐FLP dual‐active site in Pt_SA_‐(100)CeO_2_ via the synergistic effect of Pt_SA_‐Ce^3+^ and the neighboring surface lattice oxygen. Meanwhile, the atom distance and the adsorption method of Pt_SA_ in Pt_SA_‐(111)CeO_2_ inhibit the formation of FLP. The N_2_ adsorption‐desorption isotherms of various samples are shown in Figures S14 and S15 (Supporting Information), which confirms the introduction of Pt_SA_ in (100)CeO_2_ can inhibit the phenomenon of specific surface area reduction caused by high‐temperature treatment.

### 2.2 Gas Performance of Detecting NO_2_ at Room Temperature

**Figure 3:**
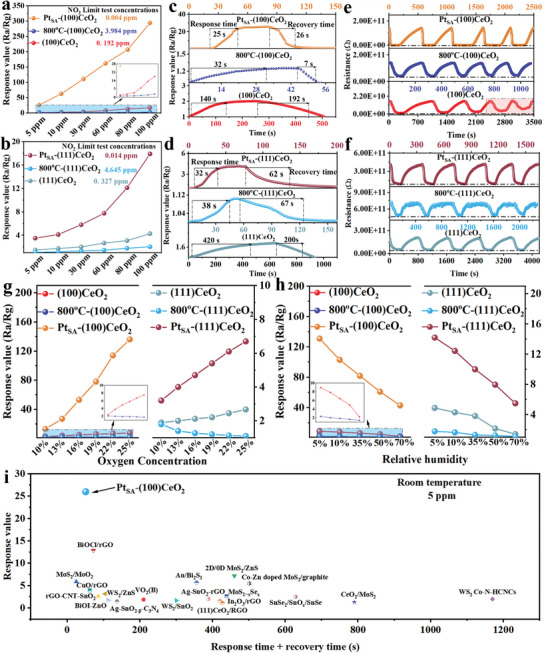
a,b) Response values of various samples toward various concentrations of NO_2_ at room temperature. c,d) Response‐recovery time of various samples toward 5 ppm NO_2_ at room temperature. e,f) Cyclic performance of various sample toward 30 ppm NO_2_ detection. g,h) The gas performance of various samples toward 30 ppm NO_2_ under different concentration oxygen molecules and different relative humidity. i) Response‐recovery time and response value toward 5 ppm NO_2_(operating temperature: 25 °C) of this work compared with previous works.

The sensing layers are estimated to be ≈80–90 µm thick (Figure S16, Supporting Information). The resistance of various samples is shown in Figure S17 (Supporting Information), which confirms the decrease of oxygen vacancy concentration, causes the resistance of CeO_2_ to increase. The response values of (100)CeO_2_, 800 °C‐(100)CeO_2_, and Pt_SA_‐(100)CeO_2_ toward various concentrations of NO_2_ at room temperature are shown in **Figure** **3**a. The response value of (100)CeO_2_ toward NO_2_ detection is higher than 800 °C‐(100)CeO_2_ due to the variability in surface oxygen vacancies and Ce^3+^ concentration. The gas performance of Pt_SA_‐(100)CeO_2_ is significantly better than (100)CeO_2_. The introduction of Pt_SA_ induces Pt_SA_‐FLP dual‐active site of (100)CeO_2_ surface, further enhancing the response value. The gas‐sensing performance of (111)CeO_2_, 800 °C‐(111)CeO_2_ and Pt_SA_‐(111)CeO_2_ toward various concentrations of NO_2_ at room temperature is shown in Figure 3b. Similarly, the response value of (111)CeO_2_ toward NO_2_ detection is higher than 800 °C‐(111)CeO_2_, and the response value of Pt_SA_‐(111)CeO_2_ toward NO_2_ detection is also higher than (111)CeO_2_. The geometry of (111)CeO_2_ is higher than that of (100)CeO_2_, indicating that the surface energy of (111)CeO_2_ is much lower than that of (100)CeO_2_. The addition of Pt_SA_ considerably only alters the surface electron concentration and catalytic activity of (111)CeO_2_. Additionally, the gas‐sensing performance of (100)CeO_2_ is stronger than that of (111)CeO_2_ due to the variability in surface activity. Meanwhile, the gas‐sensing performance of 800 °C‐(111)CeO_2_ is consistent with 800 °C‐(100)CeO_2_, indicating that high‐temperature treatment removes many surface active sites. The LOD values of various samples are shown in Figure S18 (Supporting Information). The LOD value of Pt_SA_‐(100)CeO_2_ is also lower than that of (100)CeO_2_ and 800 °C‐(100)CeO_2_ and the LOD value of Pt_SA_‐(111)CeO_2_ is also lower than that of (111)CeO_2_ and 800 °C‐(111)CeO_2_. The response‐recovery time of various samples is shown in Figure 3c,d. The response time of Pt_SA_‐(100)CeO_2_ toward NO_2_ detection is quicker than 800 °C‐(100)CeO_2_ due to the enhancement of the surface properties and adsorption‐activation ability. Meanwhile, the response time of 800 °C‐(100)CeO_2_ is quicker than that of (100)CeO_2_. The absence of a large number of active sites on the surface weakens the interaction between the NO_2_ and the 800 °C‐(100)CeO_2_ surface, resulting in the desorption of the NO_2_ without complete reaction on the 800 °C‐(100)CeO_2_ surface. Therefore, the recovery time of 800 °C‐(100)CeO_2_ toward NO_2_ detection is quicker than (100)CeO_2_. Particularly, the surface Ce^3+^ of Pt_SA_‐(100)CeO_2_ can also react with O_2_ and NO_2_, which causes the recovery time of 800 °C‐(100)CeO_2_ toward NO_2_ detection, which is also quicker than Pt_SA_‐(100)CeO_2_. Fairly, the response‐recovery time of Pt_SA_‐(111)CeO_2_ toward NO_2_ detection is quicker than 800 °C‐(111)CeO_2_ due to the enhancement of adsorption‐activation ability and the response‐recovery time of 800 °C‐(111)CeO_2_ is quicker than that of (111)CeO_2_. The sensing performance of (100)CeO_2_, 800 °C‐(100)CeO_2_ and Pt_SA_‐(100)CeO_2_ are tested toward 30 ppm NO_2_ at room temperature for five response‐recovery cycles. As shown in Figure 3e, the sensing performance of 800 °C‐(100)CeO_2_ and Pt_SA_‐(100)CeO_2_ exhibited a superior long‐term baseline resistance stability with quickly response‐recovery time. However, the gas‐sensing performance of (100)CeO_2_ significantly deteriorated due to the decrease in oxygen vacancies and Ce^3+^ concentration. Therefore, the service life and stability of the Pt_SA_‐(100)CeO_2_ are enhanced by fixing the unstable active site on the (100)CeO_2_ surface with the stable active site of Pt_SA_. Additionally, high‐temperature treatment increases the crystallinity of (100)CeO_2_ and reduces the surface energy, so 800 °C‐(100)CeO_2_ is more stable and less susceptible to O_2_ and NO_2_. The gas‐sensing stability ability of (111)CeO_2_, 800 °C‐(111)CeO_2_ and Pt_SA_‐(111)CeO_2_ are shown in Figure 3f. The sensing performance of (111)CeO_2_, 800 °C‐(111)CeO_2_, and Pt_SA_‐(111)CeO_2_ exhibited long‐term baseline resistance stability with quick response‐recovery time due to the higher geometry and lower surface energy of (111)CeO_2_, which decrease the influence of O_2_ and NO_2_.

The baseline resistance of various sample toward various concentrations of O_2_ is tested to explore the variability in surface formation activity oxygen ability of various samples. As shown in Figure S19 (Supporting Information), the baseline resistance of (100)CeO_2_, 800 °C‐(100)CeO_2_, and Pt_SA_‐(100)CeO_2_ are increased with the increase of oxygen concentration. The resistance of Pt_SA_‐(100)CeO_2_ changes much more than that of (100)CeO_2_ and 800 °C‐(100)CeO_2_, indicating that the introduction of Pt_SA_‐FLP dual‐active site enhanced the activation ability of (100)CeO_2_ to oxygen molecules. The baseline resistance of (111)CeO_2_, 800 °C‐(111)CeO_2_, and Pt_SA_‐(111)CeO_2_ are also increased with the increase of oxygen concentration. However, the resistance change degree of Pt_SA_‐(111)CeO_2_ is slightly greater than that of (111)CeO_2_. The reaction mechanism between activity species and oxygen molecules are further explored via testing the gas sensing performance at different oxygen concentrations. As shown in Figure 3g, the response value of (100)CeO_2_ and Pt_SA_‐(100)CeO_2_ toward NO_2_ detection are increased with the increase of oxygen concentrations. Particularly, the variation in response value of Pt_SA_‐(100)CeO_2_ is significantly stronger than that of (100)CeO_2_ due to the enhancement of activity oxygen concentration. The response value of 800 °C‐(100)CeO_2_ toward NO_2_ detection are decreased with the increase of oxygen concentrations, confirming that the surface reaction site could not satisfy the simultaneous adsorption of NO_2_ and O_2_, resulting in the formation of competitive adsorption of NO_2_ and O_2_ on the surface. The response value of (111)CeO_2_ and Pt_SA_‐(111)CeO_2_ toward NO_2_ detection are also increased with the increase of oxygen concentrations and the response value of 800 °C‐(111)CeO_2_ toward NO_2_ detection are decreased with the increase of oxygen concentrations. However, the variation in response value of Pt_SA_‐(111)CeO_2_ is significantly lower than that of Pt_SA_‐(100)CeO_2_, indicating that the different surface coordination modes of Pt_SA_ affect the surface electron distribute structure of Lewis acid and Lewis bases, thus changing the adsorption state of cerium oxide for oxygen and NO_2_. In general, humidity can influence the gas‐sensing performance of MOS as the operating temperature is room temperature. As shown in Figure 3h, the response value of various samples toward NO_2_ detection are decreased with the increase of relative humidity at room temperature. Particularly, the introduction of Pt_SA_ can promote the adsorption of Pt_SA_‐(100)CeO_2_ and Pt_SA_‐(111)CeO_2_ for water molecules, causing the variation in response value of Pt_SA_‐(100)CeO_2_ and Pt_SA_‐(111)CeO_2_ are significantly stronger than that of other samples. Particularly, the formation of Pt_SA_‐FLP dual activity site further enhanced the adsorption ability of (100)CeO_2_ to water molecules, causing the response value of Pt_SA_‐(100)CeO_2_ decreased significantly more than that of Pt_SA_‐(111)CeO_2_. The baseline resistance and the resistance transition of various samples toward 30 ppm NO_2_ in different relative humidity are shown in Figures S20 and S25 (Supporting Information). The resistance of various samples decreases with the increase in relative humidity. Since the water molecules can generate protons by ionization, which can produce the H+ and improve electrical conductivity. Thus, the resistance of various samples is decreased with the increase in relative humidity.

The selectivity of various samples is shown in Figure S26 (Supporting Information). At 10 ppm concentration of various target gas molecules, the response value for detecting NO_2_ is marginally better than for detecting CO and NH3 at room temperature due to the simple traditional electron transfer and surface adsorption‐reaction processes between the sample and target gas. The formation of Pt_SA_‐FLP dual activity site enhances the long‐term stability of Pt_SA_‐(100)CeO_2_ compared with (100)CeO_2_ (Figures S27 and S28, Supporting Information). In order to verify the practical application of the sensors, (100)CeO_2_, (111)CeO_2_, 800 °C‐(100)CeO_2_, 800 °C‐(111)CeO_2_, Pt_SA_‐(100)CeO_2_, and Pt_SA_‐(111)CeO_2_ are not kept in a closed dry environment during the long‐term stability test. Therefore, water molecules and other interfering gases can be adsorbed on the sensor surface to occupy the adsorption site and react with the sensitive layer, decreasing the concentration of Ce3+ and oxygen vacancies. Thus, the long‐term stability of (100)CeO_2_, (111)CeO_2_, 800 °C‐(100)CeO_2_, 800 °C‐(111)CeO_2_, Pt_SA_‐(100)CeO_2_, and Pt_SA_‐(111)CeO_2_ are decreased. The response value of Pt_SA_‐(100)CeO_2_ to 50‐ppb concentration of NO_2_, which also reaches 2.5 (Figure S29, Supporting Information). This response value and response‐recovery time of Pt_SA_‐(100)CeO_2_ is superior to previous studies for detecting NO_2_ using a semiconductor‐based gas sensor (Figure 3i; Table S1, Supporting Information). The structure stability of Pt_SA_‐(100)CeO_2_ is further explored via XPS spectra, As shown in Figure S30 (Supporting Information), The XPS spectra of Ce and O in Pt_SA_‐(100)CeO_2_ after reaction confirm the concentration of Ce^3+^ and oxygen vacancies in Pt_SA_‐(100)CeO_2_ have good stability.

### 2.3 Surface Reaction Mechanism and Density Functional Theory Calculations

**Figure 4:**
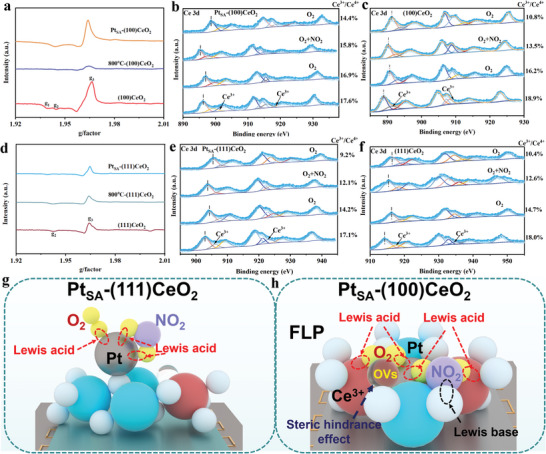
a) EPR spectra of (100)CeO_2_, 800 °C‐(100)CeO_2_ and Pt_SA_‐(100)CeO_2_. b,c) In situ NAP‐XPS Ce 3d of Pt_SA_‐(100)CeO_2_ and (100)CeO_2_ recorded at room temperature after exposed to O_2_, NO_2_+O_2_ and O_2_. d) EPR spectra of (111)CeO_2_, 800 °C‐(111)CeO_2_, and Pt_SA_‐(111)CeO_2_. e,‐f) In situ NAP‐XPS Ce 3d of Pt_SA_‐(111)CeO_2_ and (111)CeO_2_ recorded at room temperature after exposed to O_2_, NO_2_+O_2_ and O_2_. g,‐h)The surface reaction schematic illustration of Pt_SA_‐(100)CeO_2_ and Pt_SA_‐(111)CeO_2_ toward NO_2_.

The Electron Paramagnetic Resonance (EPR) spectra of Pt_SA_‐(100)CeO_2_, 800 °C‐(100)CeO_2_, and (100)CeO_2_ are shown in **Figure** **4**a, there are three different signals of g value in the EPR spectra. The (g1,g3) and (g2,g3) are linked with low coordination Ce^3+^ and high coordination Ce^3+^, which can provide electron reaction with oxygen molecules to generate activity‐oxygen. Due to the high temperature heat treatment, the low coordination Ce^3+^ disappeared, and the high coordination Ce^3+^ intensity of (100)CeO_2_ is stronger than that of Pt_SA_‐(100)CeO_2_ and 800 °C‐(100)CeO_2_. While the high coordination Ce^3+^ intensity of Pt_SA_‐(100)CeO_2_ is stronger than that of 800 °C‐(100)CeO_2_. The introduction of Pt_SA_ can maintain the Ce^3+^ activity site, which promotes the adsorption‐activation ability toward O_2_ and NO_2_. The surface reaction mechanism of Pt_SA_‐(100)CeO_2_, 800 °C‐(100)CeO_2_ and (100)CeO_2_ are further explored via in situ NAP‐XPS. As shown in Figure 4b,c, Pt_SA_‐(100)CeO_2_ is activated at room temperature and then exposed to O_2_, the spectra shift to higher binding energy, indicating that the electron is transferred from Pt_SA_‐(100)CeO_2_ to O_2_ and produces activity oxygen. Additionally, the Ce^3+^ concentration of Pt_SA_‐(100)CeO_2_ decreased from 17.6% to 16.9%, while the Ce^3+^ concentration of (100)CeO_2_ decreased from 18.9% to 16.2%. Then, exposing Pt_SA_‐(100)CeO_2_ to a mixture of NO_2_ and O_2_ causes the spectra to shift to lower binding energy due to the reaction between NO_2_ and activity oxygen. The Ce^3+^ concentration of Pt_SA_‐(100)CeO_2_ has decreased from 16.9% to 15.8%, and the Ce^3+^ concentration of (100)CeO_2_ has decreased from 16.2% to 13.5%. Finally, exposing Pt_SA_‐(100)CeO_2_ to O_2_ causes the Ce^3+^ concentration to decrease from 15.8% to 14.4%, while the Ce^3+^ concentration of (100)CeO_2_ is decreased from 13.5% to 10.8%. In situ NAP‐XPS O 1s of Pt_SA_‐(100)CeO_2_ and (100)CeO_2_ are shown in Figures S31 and S32 (Supporting Information). Therefore, compared with (100)CeO_2_, the Pt_SA_ occupies the four‐fold hollow sites on the (100)CeO_2_ surface to maintain the Ce^3+^ and oxygen vacancies concentration and change the adsorption method toward O_2_ and NO_2_ during the reaction process. The EPR spectra of Pt_SA_‐(100)CeO_2_, 800 °C‐(100)CeO_2_ and (100)CeO_2_ are shown in Figure 4d. The Ce^3+^ concentration of (111)CeO_2_ is significantly lower than that of (100)CeO_2_, while the Ce^3+^ stability of (111)CeO_2_ is stronger than that of (100)CeO_2_. Additionally, the concentration of Ce^3+^ on (111)CeO_2_ surface is not affected by the introduction of Pt_SA_ into (111)CeO_2_, which further confirms the distinction of Pt_SA_ Chemical coordination at (111)CeO_2_ and (100)CeO_2_. Similarly, in situ NAP‐XPS spectra of Pt_SA_‐(111)CeO_2_ and (111)CeO_2_ are shown in Figure 4e,f. Pt_SA_‐(111)CeO_2_ is activated at room temperature and then exposed to O_2_, the Ce^3+^ concentration of Pt_SA_‐(111)CeO_2_ is decreased from 17.1% to 14.2%, and the Ce^3+^ concentration of (111)CeO_2_ decreases from 18.0% to 14.7%. Then, exposing Pt_SA_‐(111)CeO_2_ to a mixture of NO_2_ and O_2_ caused Ce^3+^ concentration to decrease from 14.2% to 12.1%, and Ce^3+^ concentration of (111)CeO_2_ decreased from 14.7% to 12.6%. Finally, exposing Pt_SA_‐(111)CeO_2_ to O_2_ caused the Ce^3+^ concentration to decrease from 12.1% to 9.2%, and the Ce^3+^ concentration of (111)CeO_2_ decreased from 12.6% to 10.4%. In situ Near Ambient Pressure XPS (NAP‐XPS) O 1s of Pt_SA_‐(111)CeO_2_ and (111)CeO_2_ are shown in Figures S33 and S34 (Supporting Information). Hence, the schematic diagram of O_2_ and NO_2_ on the surface of Pt_SA_‐(111)CeO_2_ and Pt_SA_‐(100)CeO_2_ for the adsorption‐activation method is shown in Figure 4g,h. No electron interaction exists between Pt_SA_ and Ce^3+^ on the surface of Pt_SA_‐(111)CeO_2_, separated active sites that adsorb and activate O_2_ and NO_2_, respectively. Inversely, there is a strong electronic interaction between Pt_SA_ and Ce^3+^ on the surface of Pt_SA_‐(100)CeO_2_ due to the quadruple hollow site occupied by Pt_SA_. Additionally, the construction of FLP formed a double active site, causing the joint adsorption and activation of O_2_ and NO_2_ by Pt_SA_ and Ce^3+^, which speeds up the transfer efficiency of electrons between the target molecule and Pt_SA_‐(100)CeO_2_.

**Figure 5:**
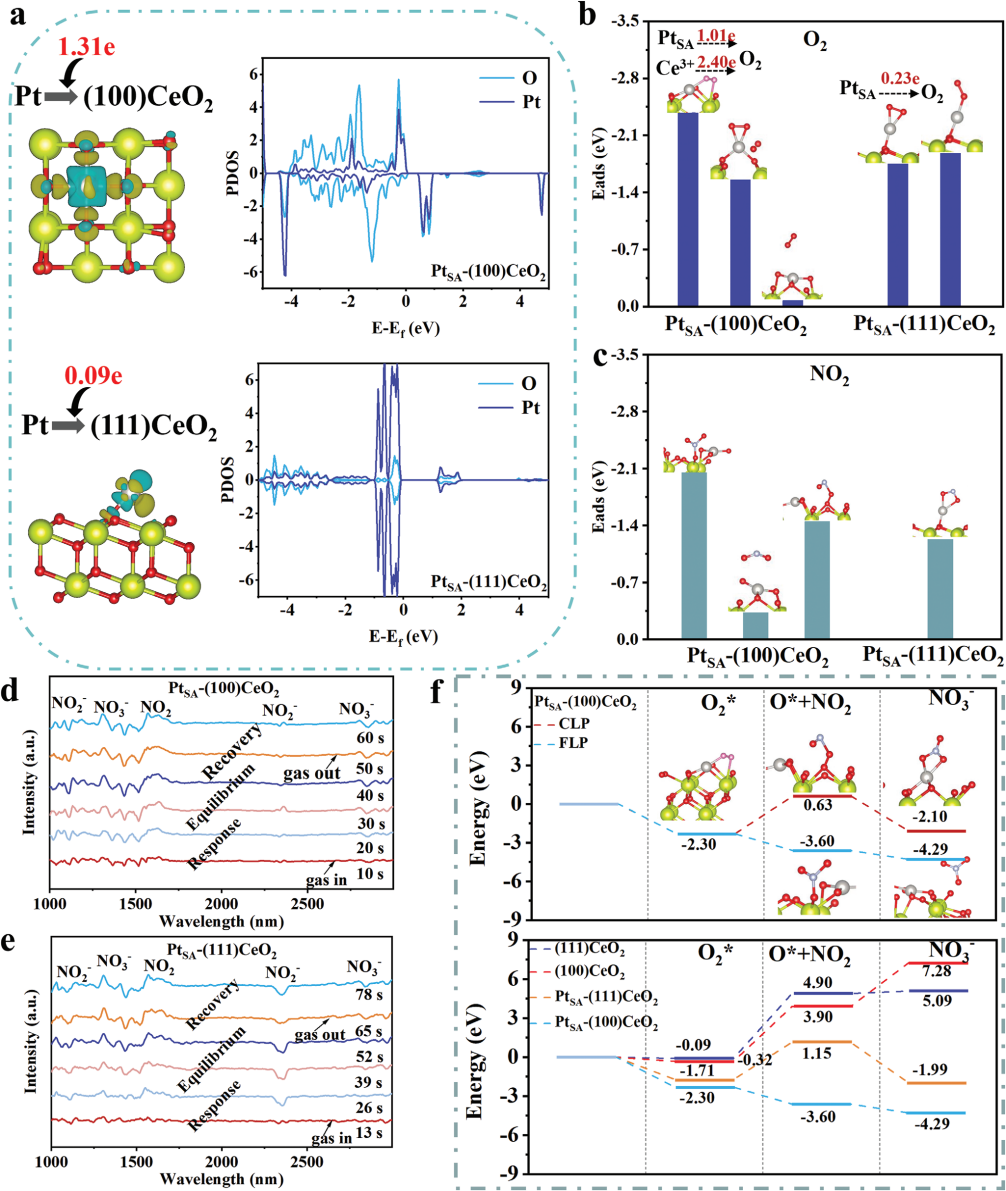
a) Charge difference distributions and DOS of Pt_SA_‐(100)CeO_2_ and Pt_SA_‐(111)CeO_2_. b,c) The adsorption model of Pt_SA_‐(100)CeO_2_ and Pt_SA_‐(111)CeO_2_ toward O_2_ and NO_2_. d,e) In situ FTIR spectra during gas‐sensing reaction on the surface of Pt_SA_‐(100)CeO_2_ and Pt_SA_‐(111)CeO_2_ toward NO_2_ under different test time. f) Activation energies (*E*_a_) for the reaction of NO_2_ with activity oxygen on various samples.

The surface electronic structures and adsorption‐reaction sites of Pt_SA_‐(100)CeO_2_ and Pt_SA_‐(111)CeO_2_ are explored via DFT calculations. As shown in **Figures** **5**a and S35 (Supporting Information), Bader charge analysis confirms that Pt_SA_ transfers 1.31e to (100)CeO_2_ and Pt_SA_ transfers 0.09e to (111)CeO_2_. Additionally, compared with the partial density of states (PDOS) of Pt_SA_‐(111)CeO_2_, the introduction of Pt_SA_ in (100)CeO_2_ caused the presence of Pt 5d‐states at the Fermi level and asymmetric distribution of DOS, resulting in improvement in electronic properties. The PDOS of Ce and O are shifted toward the fermi level after Pt adsorption on (100)CeO_2_. Meanwhile, for (111)CeO_2_, the PDOS shifted away from the Fermi level. Figure 5b shows adsorption‐activation ability of O_2_ on the surface of Pt_SA_‐(111)CeO_2_ and Pt_SA_‐(100)CeO_2_. The adsorption model of O_2_ on the surface of Pt_SA_‐(100)CeO_2_ is divided into parallel adsorption (the adsorption site is Pt_SA_ or Pt_SA_ and Ce^3+^) and vertical adsorption (the adsorption site is Pt_SA_). The adsorption energies for O_2_ of Pt_SA_‐(100)CeO_2_ are −1.52, −2.35, and −0.11 eV, indicating the surface of Pt_SA_‐(100)CeO_2_ is more inclined to the co‐adsorption of Pt_SA_ and Ce^3+^ toward O_2_. The adsorption energies for O_2_ of Pt_SA_‐(111)CeO_2_ are −1.62 eV (parallel adsorption) and −1.71 eV (vertical adsorption). The adsorption energies for O_2_ of (100)CeO_2_ and (111)CeO_2_ are −0.32 and −0.08 eV (Figure S36, Supporting Information). Figure 5c shows adsorption‐activation ability of NO_2_ on the surface of Pt_SA_‐(111)CeO_2_ and Pt_SA_‐(100)CeO_2_. The adsorption method of NO_2_ on the surface of Pt_SA_‐(100)CeO_2_ is divided into FLP and Classic Lewis acid‐base pair (CLP). The adsorption energies for NO_2_ of Pt_SA_‐(100)CeO_2_ are −2.08 eV (FLP), −0.31 eV (CLP), and −1.44 eV (CLP, O‐OVs). The adsorption energies for NO_2_ of Pt_SA_‐(111)CeO_2_ are −1.23 eV. The adsorption energies for NO_2_ of (100)CeO_2_ and (111)CeO_2_ are −0.19 and −0.12 eV (Figure S37, Supporting Information).

The in situ FTIR spectroscopy is conducted to analyze NO_2_ reaction on the surface of various samples at room temperature. As shown in Figure 5d, the surface reaction process of Pt_SA_‐(100)CeO_2_ toward NO_2_ detection can be divided to three parts. The peaks observed at 1589 cm^−1^ correspond to the surface‐adsorbed NO_2_ and the absorbance intensity of NO_2_ increases during the response process.^[^ ^33^ ^]^ At the same time, the peaks at 1136 and 2361 cm^−1^ correspond to the generation of NO_2_^−^ and the intensity of NO_2_^−^ also increases.^[^ ^34^ ^]^ The peaks at 1307 and 2854 cm^−1^ correspond to the generation of mo‐NO_3_^−^ and the intensity of br‐NO_3_^−^ also increases.^[^ ^35^ ^]^ Then, the peak intensity of adsorb NO_2_, NO_2_^−^ and NO_3_^−^ reach equilibrium during the reaction equilibrium process. During recovery, the absorbance intensity of NO_2_^−^ decreases, and the peak intensity corresponding to the NO_3_^−^ increases. The result confirms that the NO_2_^−^ can react with •O_2_^−^ to produce NO_3_^−^ during the recovery process. The in situ FTIR spectra of (100)CeO_2_ and 800 °C‐(100)CeO_2_ confirmed that high temperatures destroy the surface activity, resulting in a tendency of (100)CeO2 to produce NO_2_^−^ (Figure S38, Supporting Information). The surface reaction process of Pt_SA_‐(111)CeO_2_ is significantly changed compared with Pt_SA_‐(100)CeO_2_. At the response and equilibrium processes, the peaks at 1137 and 2350 cm^−1^ correspond to the generation of NO_2_^−^ and the peak intensity increases.^[^ ^36^ ^]^ The peak at 1303 cm^−1^ corresponds to the generation of mo‐NO_3_^−^, and the peak intensity also increases. At the recovery process, the peak at 2817 cm^−1^ corresponds to the br‐NO_3_^−^ is presented, which is originally from the reaction between NO_2_^−^ and •O_2_^−^.^[^ ^37^ ^]^ Therefore, Pt_SA_‐(111)CeO_2_ is more inclined to produce NO_2_^−^ and Pt_SA_‐(100)CeO_2_ is more inclined to produce NO_3_^−^, which means that the activation ability of Pt_SA_‐(100)CeO_2_ surface to NO_2_ is stronger than that of Pt_SA_‐(111)CeO_2_ surface to NO_2_. The in situ FTIR spectra of (111)CeO_2_ and 800 °C‐(111)CeO_2_ are shown in Figure S39 (Supporting Information). The 2D pseudo‐color in situ FTIR spectra of various samples are shown in Figures S40 and S41 (Supporting Information), which further confirms the change of surface products. Based on the analysis of in ‐situ FTIR spectra result and adsorption energy, the final products and reaction models of (111)CeO_2_, (100)CeO_2_, Pt_SA_‐(111)CeO_2_ and Pt_SA_‐(100)CeO_2_ toward NO_2_ detection process are determined. The activation barriers of CLP and FLP in Pt_SA_‐(100)CeO_2_ surface are further explored via DFT calculations. As shown in Figure 5f, the oxygen molecules can first react with photo‐generating electrons to spontaneously generate •O_2_^−^ species on the surface of Pt_SA_‐(100)CeO_2_. Then, the NO2 can be adsorbed and react with •O_2_^−^ species to generate NO_3_^−^. The reaction barriers of CLP and FLP toward NO2 adsorption are 0.63 eV and −3.60 eV, indicating that the construction of FLP enhances the activation ability of Pt_SA_‐(100)CeO_2_ toward NO_2_. Additionally, the reaction barriers of Pt_SA_‐(100)CeO_2_ is lower than that of (100)CeO_2_, (111)CeO_2_ and Pt_SA_‐(111)CeO_2_ during the reaction between O_2_ and electron process. Then, the reaction barriers of Pt_SA_‐(100)CeO_2_, (100)CeO_2_, (111)CeO_2_ and Pt_SA_‐(111)CeO_2_ toward the process of NO_2_ convert into NO_3_^−^ are −3.60, 3.90, 1.15, and 4.90 eV, indicating that the construction of SA‐FLP dual active sites promote the activation and reaction of NO_2_ and O_2_ on the Pt_SA_‐(100)CeO_2_ surface. Hence, the dramatic fast and long‐term stability detection of NO_2_ results from the synergy of SA‐FLP dual active sites.

## 3 Conclusion

In this work, we explore the evolution of Pt_SA_ on (100)CeO_2_ and (111)CeO_2_, revealing notable disparities in the position and activity of surface Pt_SA_ on different crystal planes. The AC‐TEM and in situ NAP‐XPS analysis confirm that Pt_SA_ occupies the quadruple hollow site of (100)CeO_2_ surface and improves the stability of Ce^3+^ and oxygen vacancies. Notably, the Pt_SA_ induces surface reorganization and combines the steric hindrance effect of oxygen vacancies to construct an FLP to form a double‐active site. The DFT calculation confirms that the adsorption ability of O_2_ and NO_2_ is efficiently enhanced with the help of Lewis acidic Ce^3+^ sites of FLPs. The FLP sites constructed on Pt_SA_‐(100)CeO_2_ promote the O_2_ and NO_2_ activation, producing abundant surface activity oxygen for the subsequent transformation of NO_2_ and activity oxygen into NO_3_^−^. Conversely, Pt_SA_ tends to bind to terminal oxygen on the surface of (111)CeO_2_ and become an independent reaction site, which has limited performance improvement in detecting NO_2_. This research underscores the correlation between single atoms and crystal plane effects, laying the groundwork for designing and synthesizing ultra‐stable and efficient gas sensors.

## Conflict of Interest

The authors declare no conflict of interest.

## Supporting information

Supporting Information

## Data Availability

The data that support the findings of this study are available from the corresponding author upon reasonable request.
