# Supplementary material for: Crystal Face‐Dependent Behavior of Single‐Atom Pt: Construct of SA‐FLP Dual Active Sites for Efficient NO2 Detection
Source: Adv Sci (Weinh). 2024 May 29;11(29):2402038. doi: 10.1002/advs.202402038 (PMC11304280; doi:10.1002/advs.202402038)
Supplement: Supplementary file 1 — Supporting Information [file ADVS-11-2402038-s001.docx]

**Crystal face-dependent behavior of single-Atom Pt: Construct of SA-FLP dual active sites for efficient NO_2_ detection**

Yucheng Ou^a^, Bing Wang^a*^, Nana Xu^a^, Quzhi Song^a^, Tao Liu^a^, Hui Xu^a^, Fuwen Wang^a^, Yingde Wang^a^*

*^a^Science and Technology on Advanced Ceramic Fiber and Composites Laboratory, College of Aerospace Science and Engineering,* *National University of Defense Technology, Changsha 410073, China*

**Corresponding authors:*

*E-Mail: wangyingde@nudt.edu.cn*

*bingwang@nudt.edu.cn*

**Contents**

**I. Methods**

**II. Supplementary Tables**

**Tables S1**

**III. Supplementary Figures**

**Figs. S1 to S41**

**I. Methods**

**1. Chemicals and Materials.** Tetraammineplatinum(II) nitrate (H_12_N_6_O_6_Pt, 99.99%), and Cerous nitrate hexahydrate (Ce(NO_3_)_3_·6H_2_O, 99.95%) were purchased from Aladdin, sodium hydroxide (NaOH, ≧98%)were purchased from Aladdin. The water used in all experiments was ultrapure (18.2 Ω/cm).

**2. Experimental Section**

**2.1 Synthesis of (111)CeO_2_.** (111)CeO_2_ was synthesized by hydrothermal Method. 0.868 g Ce(NO_3_)_3_·6H_2_O and 0.016 g NaOH are dissolved in 35 mL and 5 ml of distilled water, respectively. As the clear solution formed, NaOH aqueous solution is slowly added to the Ce(NO_3_)_3_·6H_2_O with vigorous stirring. After stirring for about 30 min, the mixture solution was subjected to hydrothermal treatment at 180 ^o^C for 24 h. The obtained powders were washed with water and ethanol for three cycles, dried in vacuum over a night, and further subjected to calcination at 550 ^o^C for 4 h.

**2.2 Synthesis of (100)CeO_2_.** (100)CeO_2_ was synthesized by hydrothermal method as well. 0.868 g Ce(NO_3_)_3_·6H_2_O and 9.6 g NaOH are dissolved in 35 mL and 5 ml of distilled water, respectively. As the clear solution formed, NaOH aqueous solution is slowly added to the Ce(NO_3_)_3_·6H_2_O with vigorous stirring. After stirring for about 30 mins, and mixture solution was subjected to hydrothermal treatment at 180 ^o^C for 24 h. The obtained powders were washed with water and ethanol for three cycles, dried in vacuum over a night, and further subjected to calcination at 550 ^o^C for 4 h.

**2.3 Synthesis of** **800^o^C-(111)CeO_2_ and 800^o^C-(100)CeO_2_.** 800^o^C-(111)CeO_2_ and 800^o^C-(100)CeO_2_ are synthesized by the high temperature heat treatment method. 0.1 g (111)CeO_2_ and (100)CeO_2_ are heat treatment at 800^o^C for 10 h under flowing dry air.

**2.4 Synthesis of** **Pt_SA_-(111)CeO_2_ and Pt_SA_-(100)CeO_2_.** Pt_SA_-(111)CeO_2_ and Pt_SA_-(100)CeO_2_ are synthesized by the high temperature heat treatment method. 0.1 g (111)CeO_2_ and (100)CeO_2_ are dispersed in 30 ml of distilled water in stirring, respectively. As the powder disperses evenly, 0.2wt% of Pt solution (H_12_N_6_O_6_Pt) was added. Then, the mixed solution was stirring for 12 h, the powder was collected washed. Finally, the powder was heat treatment at 800^o^C for 10 h under flowing dry air.

**3. Characterization and measurements.** X-ray diffraction (XRD) patterns were recorded on a Bruker D8 X-ray powder diffractometer with Cu Kα radiation (λ = 1.5418 Å) at 30 kV and 10 mA with a scanning rate of 5° min^-1^ in the 2θ range of 10° ~ 80°. The HRTEM images were taken on a Talos F200i working at 200 kV and JEOL JEM-2100F field emission transmission electron microscopy with an accelerating voltage of 200 kV. The high-angle annular dark-field scanning transmission electron microscopy (HAADF-STEM) images and Xray energy dispersive spectroscopy (EDS) mapping was recorded on aberration-corrected TEM (FEI Titan Cubed Themis G2 300) at an accelerating voltage of 300 kV. Si (Li) EDS detector with a solid angle of 0.13 srad and Fiori number > 4000. 1.85 s/pixel and gather a 460 pixel map, so the time of EDS acquisition will cost 851 seconds. The In situ Fourier-transform infrared (In situ FT-IR) spectra were recorded on a Bruker Vertex 70 FTIR spectrometer equipped with in situ reaction chamber. X-band electron paramagnetic resonance (EPR) measurement was performed at room temperature using a JEOL FA-200 EPR spectrometer. The X-ray photoelectron spectroscopy (Axis Supra) measurements were operated with Al Ka radiation (1486.6 eV). Binding energies (BE) were calibrated by setting the measured BE of C 1s to 284.8 eV. In situ DRIFTS spectra were measured on a Nicolet-6700 FTIR spectrometer. The N_2_ adsorption-desorption isotherms were measured using a BELSORP-max-II to estimate specific surface area and pore size distribution by the Brunauer-Emmett-Teller (BET) and Barrett–Joyner–Halenda (BJH) methods.

**4. Fabrication and testing of a gas sensor and experimental detail.** 1 mg of (111)CeO_2_, (100)CeO_2_, 800^o^C-(111)CeO_2_, 800^o^C-(100)CeO_2_, (111)/(100)CeO_2_ and Pt_SA_-(100)CeO_2_ and Pt_SA_-(111)CeO_2_ were mixed with 50 μL of deionized water to obtain the corresponding slurry. 5 μL of slurry was then dripped on an Au interdigitated electrodes (10 mm × 5 mm × 0.25 mm, AURORA technologies, China) to form a resistance-type sensor. All the fabricated sensors were aged in air at 80 °C for 2 h. The gas-sensing performance of the fabricated sensors was evaluated using an intelligent gas-sensing analysis system (CGS-4TPs, Beijing Elite Tech Co., Ltd.). To produce test gases with the necessary concentrations, a dynamic gas and liquid distribution system (DGL-III, Beijing Elite Tech Co., Ltd, China) having three mass flow controllers was used. We use nitrogen as the carrier gas, and control the gas flow of oxygen and nitrogen to control the different oxygen concentrations in the reaction chamber. The interference gas is injected into the reaction chamber through a syringe. The room where the gas-sensitive analysis system is located is equipped with a constant temperature and humidity air conditioning system, and the indoor humidity can be artificially controlled by setting parameters. The response value (*S*_g_) is defined as the ratio of resistance in air (*R*_a_) to resistance in target gas (*R*_g_). The response time and recovery time are defined as the time for the sensor to reach 90% of the final signal.

**5. In-situ FTIR analysis.** The *in-situ* FTIR spectra were measured on a Bruker Vertex 70 FT-IR spectrometer equipped with an *in-situ* reaction chamber, gas system, and pretreatment equipment. Prior to the adsorption process, the sample was purged by high-purity He for 1 h at 80°C to clean the surface, and then, the baseline was obtained after cooling to 30°C. Afterwards, 15 mL·min^−1^ NO_2_ (40 ppm) and 30 mL·min^−1^ O_2_ were introduced into the reaction chamber. First, O_2_ was allowed to be adsorbed on the sample surface for 1 min. The FTIR spectra were recorded according to the response-recovery time.

**6. DFT calculations.** All the calculations were performed on the basis of the DFT methods implemented in the Vienna Ab initio Simulation Package (VASP 5.4.4). The generalized gradient approximation was used to estimate exchange–correlation interaction in the scheme of the Perdew–Burke–Ernzerhof functional. The effect of core electrons on the density of valence electrons was described using the projector augmented wave method. The kinetic energy cutoff for the plane waves was set to 450 eV for all the calculations. To consider the open-shell d-electrons, GGA+U schemes were implemented, employing effective U values of 5.0 for Ce. The convergence tolerance of energy and force on each atom during structure relaxation were less than 10^−5^ eV and 0.03 eV/Å, respectively. A set of Monkhorst–Pack mesh K points of 2×2×1 and 4×4×1 are used to sample the Brillouin zone for geometry optimization and electronic structural calculations. A vacuum distance of 15 Å was set to ensure sufficient vacuum and avoid interactions between two periods. Further calculations were carried out to determine the thermal and zero-point energy (ZPE) corrections at the Γ point of various intermediates adsorbed on the surface. The VASPKIT code was used for postprocessing computational data obtained from VASP.

The adsorption energy can be evaluated by ΔG_ads_, which is defined as

ΔE_ads_ = E_(System+ gas)_ − E_(System)_ −1/2 E _ads_,

in which E_(System+ gas)_ and E_(System)_ are the energies of all research systems with and without gas adsorption, respectively. Eads represents the energy of adsorbed intermediates.

**II. Supplementary Tables**

**Table S1.** List of NO_2_ gas sensors and their response-recovery time at room temperature toward 5ppm NO_2_.

| Material | Response time(s) | Recovery time(s) | Response valuse | Gas concentration range and low limit | References |
| --- | --- | --- | --- | --- | --- |
| CuO/rGO | 6.8 | 55.1 | 4 | 0.05 ppm-100 ppm,  0.05 ppm | Sensor Actuat B-Chem. **337,** 129783 (2021) |
| VO_2_(B) | 60 | 150 | 1.9 | 0.5 ppm-5 ppm,  0.5 ppm | ACS Appl. Mater. Interfaces. **13,** 31968-31977 (2021) |
| MoS_2_/MoO_2_ | 2 | 23 | 5.9 | 0.1 ppm-100 ppm,  0.1 ppm | Nanoscale. **11,** 8554-8564 (2019) |
| 2D/0D MoS_2_/ZnS | 60 | 400 | 7.2 | 0.05 ppm-100 ppm,  0.05 ppm | Sensor Actuat B-Chem. **347,** 130608 (2021) |
| WS_2_ Co-N-HCNCs | 570 | 600 | 2 | 0.1 ppm-1 ppm,  0.1 ppm | Adv. Funct. Mater. **28,** 1802575 (2018) |
| WS_2_/ZnS | 4 | 100 | 3.2 | 0.01 ppm-5 ppm,  0.01 ppm | Sensor Actuat B-Chem. **296,** 126666 (2019) |
| WS_2_/SnO_2_ | 200 | 100 | 1.7 | 0.5 ppm-20 ppm,  0.5 ppm | Sensor Actuat B-Chem. **364,** 131903 (2022) |
| Co-Zn doped MoS_2_/graphite | 118 | 383 | 5.5 | 0.05 ppm-5 ppm,  0.05 ppm | J Mater Chem C. **11,** 2364-2374 (2023) |
| SnSe_2_/SnO_x_/SnSe | 134 | 494 | 2.5 | 1 ppm-5 ppm,  1 ppm | J Sci-Adv Mater Dev. **8,** 100583 (2023) |
| Ag-SnO_2_-rGO | 49 | 339 | 2.1 | 1 ppm-10 ppm,  1 ppm | Sensor Actuat B-Chem. **222,** 893-903 (2016) |
| MoS_2−x_Se_x_ | 107 | 331 | 2.7 | 1 ppm-100 ppm,  1 ppm | Sensor Actuat B-Chem. **378,** 133137 (2023) |
| Ag-SnO_2-β_-C_3_N_4_ | 27 | 110 | 1.5 | 1 ppm-5 ppm,  1 ppm | Sensor Actuat B-Chem. **326,** 128910 (2021) |
| rGO-CNT-SnO_2_ | 8 | 77 | 2.53 | -,  5 ppm | Sensor Actuat B-Chem. **211,** 318-324 (2015) |
| BiOCl/rGO | 9 | 63 | 12.9 | 0.03 ppm-100 ppm,  0.03 ppm | Sensor Actuat B-Chem. **351,** 130932 (2022) |
| Au/Bi_2_S_3_ | 18 | 338 | 5.7 | 0.25 ppm-20 ppm,  0.25 ppm | ACS Sens. **7,** 816-826 (2022) |
| BiOI-ZnO | 66 | 47 | 1.7 | 0.025 ppm-1 ppm,  0.025 ppm | ACS Sens. **7,** 3915-3922 (2022) |
| In_2_O_3_/rGO | 180 | 240 | 1.6 | -,  5 ppm | ACS Appl. Mater. Interfaces. **6,** 21093-21100 (2014) |
| CeO_2_/MoS_2_ | 470 | 320 | 1.3 | 5 ppm-100 ppm,  5 ppm | Appl Surf Sci.**600,** 154157 (2022) |
| (100)CeO_2_/RGO | 189 | 1137 | 1.16 | 1 ppm-5 ppm,  1 ppm | ACS Appl. Mater. Interfaces. **12,** 4722-4731 (2020) |
| (111)CeO_2_/RGO | 181 | 246 | 1.12 | 5 ppm-200 ppm,  5 ppm | Anal. Chem. **90,** 9821-9829 (2018) |
| Pt_SA_-(100)CeO_2_ | 25 | 26 | 26 | 0.05 ppm-100 ppm,  0.05 ppm | **This work** |

**III. Supplementary Figures**


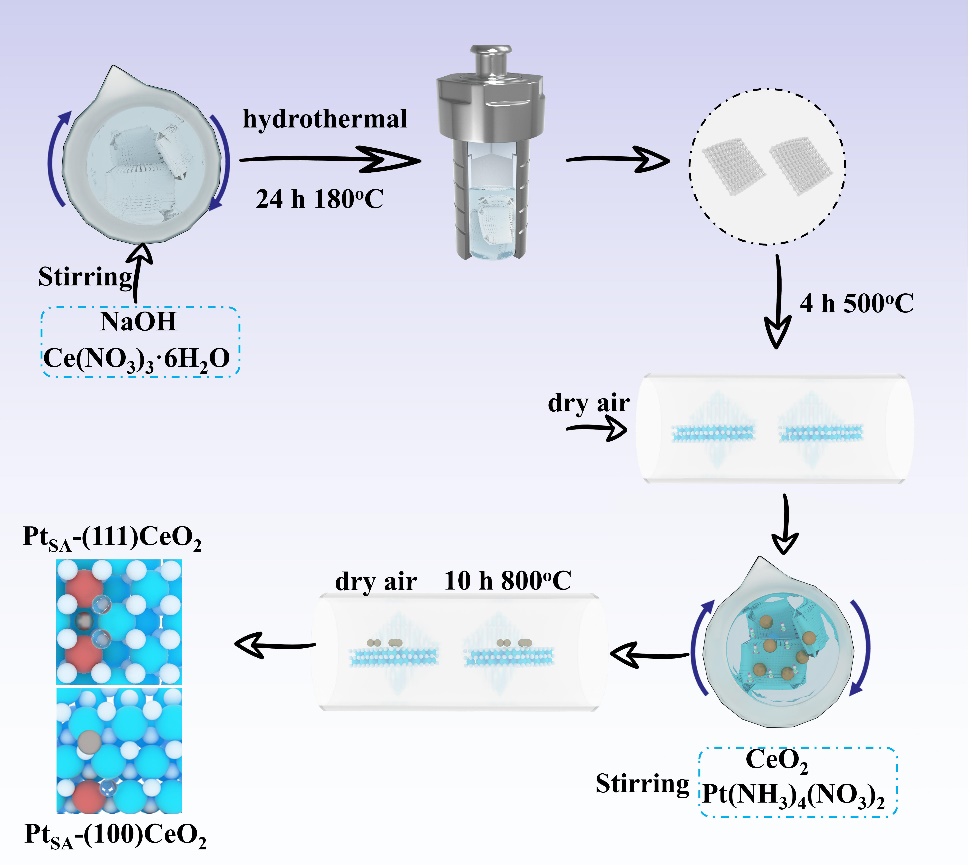


**Figure S1.** The synthesis diagrams of Pt_SA_-(111)CeO_2_ and Pt_SA_-(100)CeO_2_.


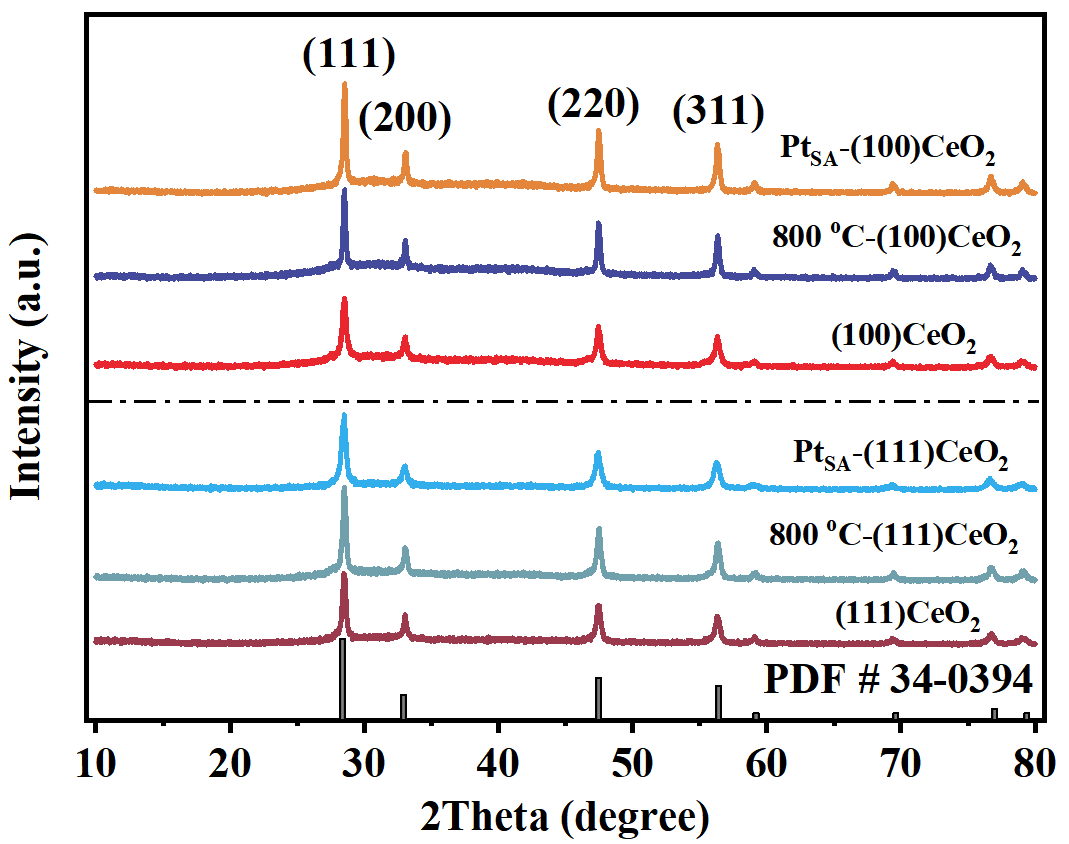


**Figure S2.** XRD patterns of (111)CeO_2_, 800^o^C-(111)CeO_2_, Pt_SA_-(111)CeO_2_ and (100)CeO_2_, 800^o^C-(100)CeO_2_, Pt_SA_-(100)CeO_2_.


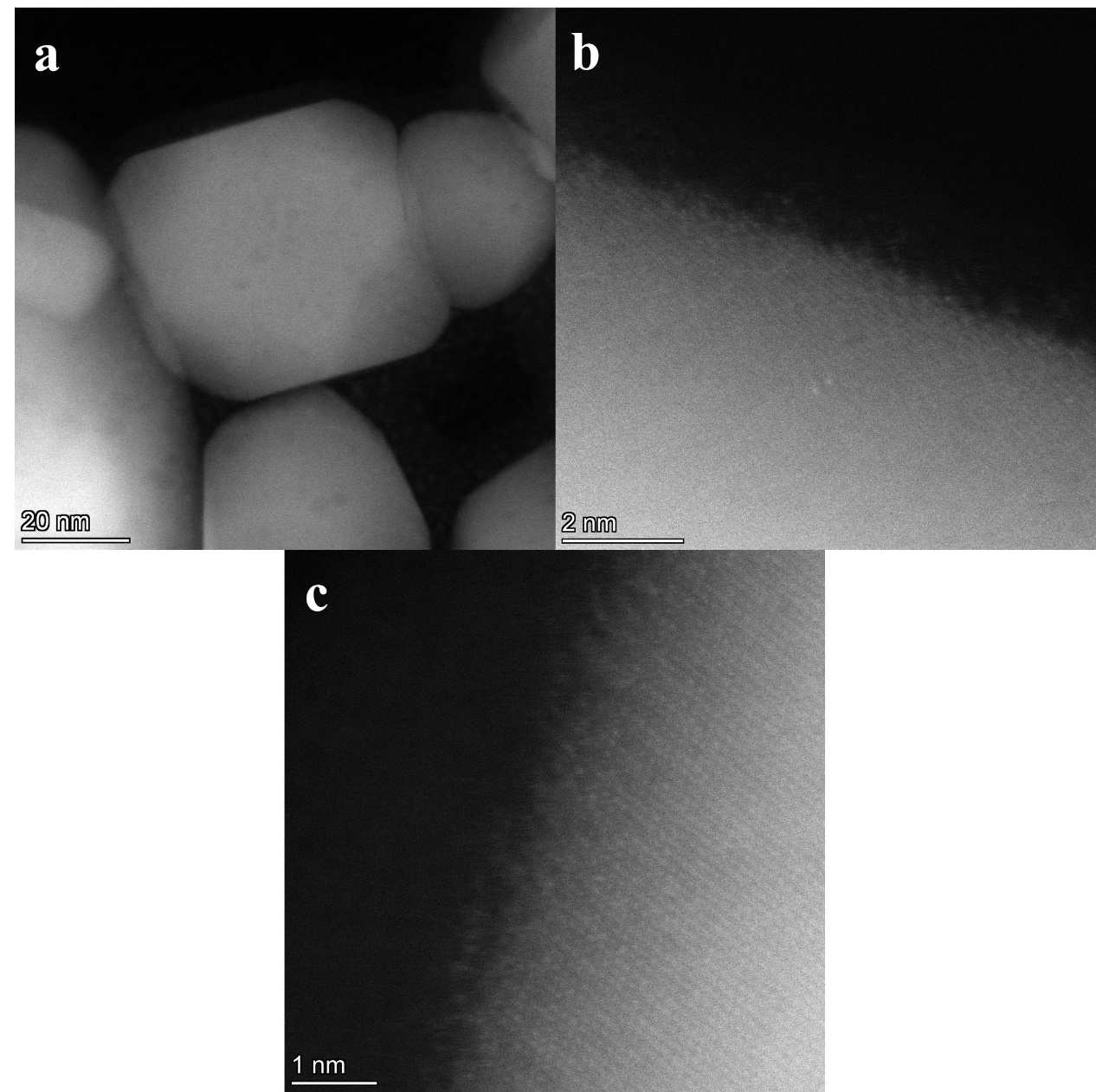


**Figure S3.** HAADF images of Pt_SA_-(100)CeO_2_.


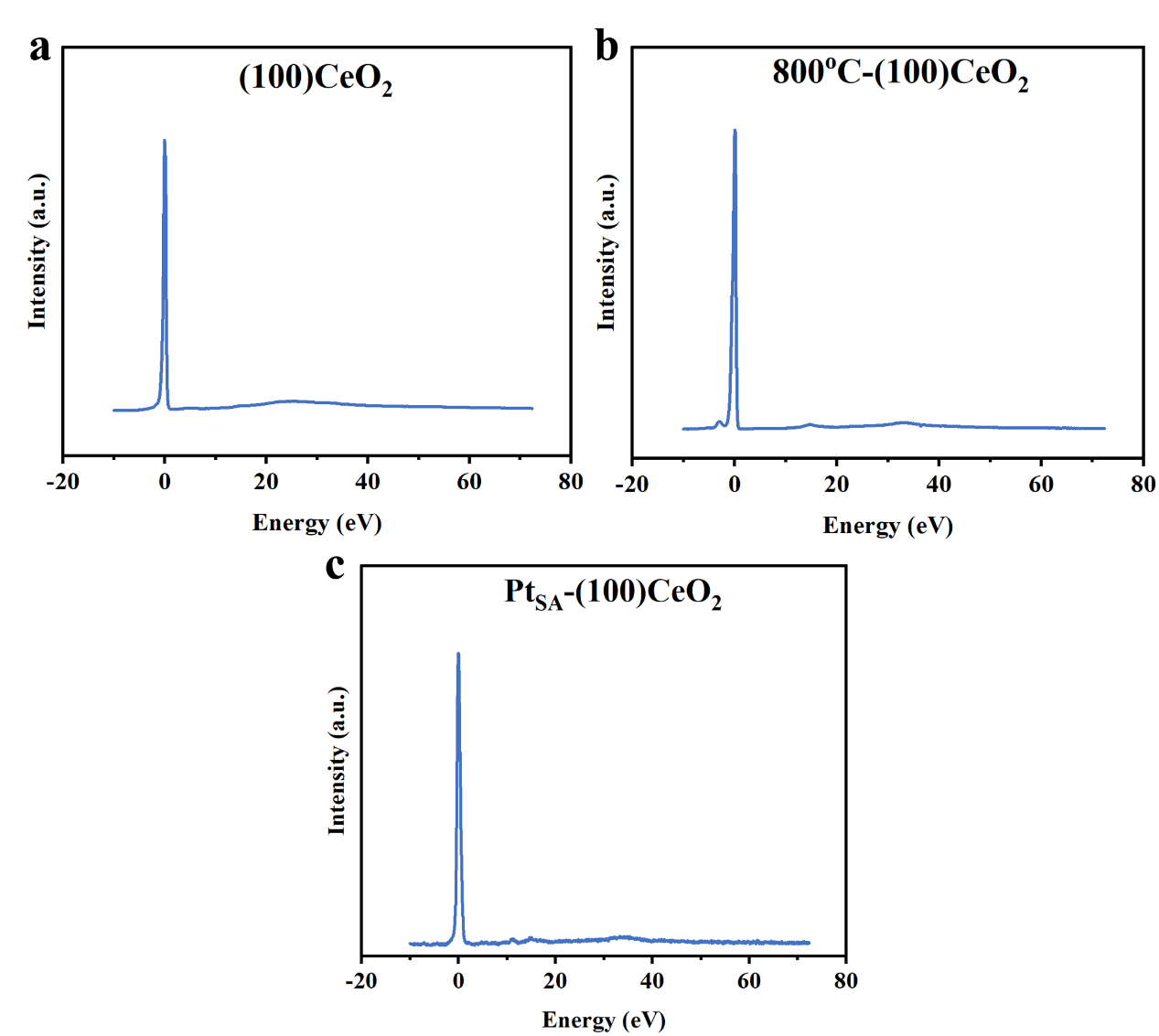
**Figure S4.** Zero-loss peak of (100)CeO_2_,800^o^C-(100)CeO_2_ and

Pt_SA_-(100)CeO_2_.


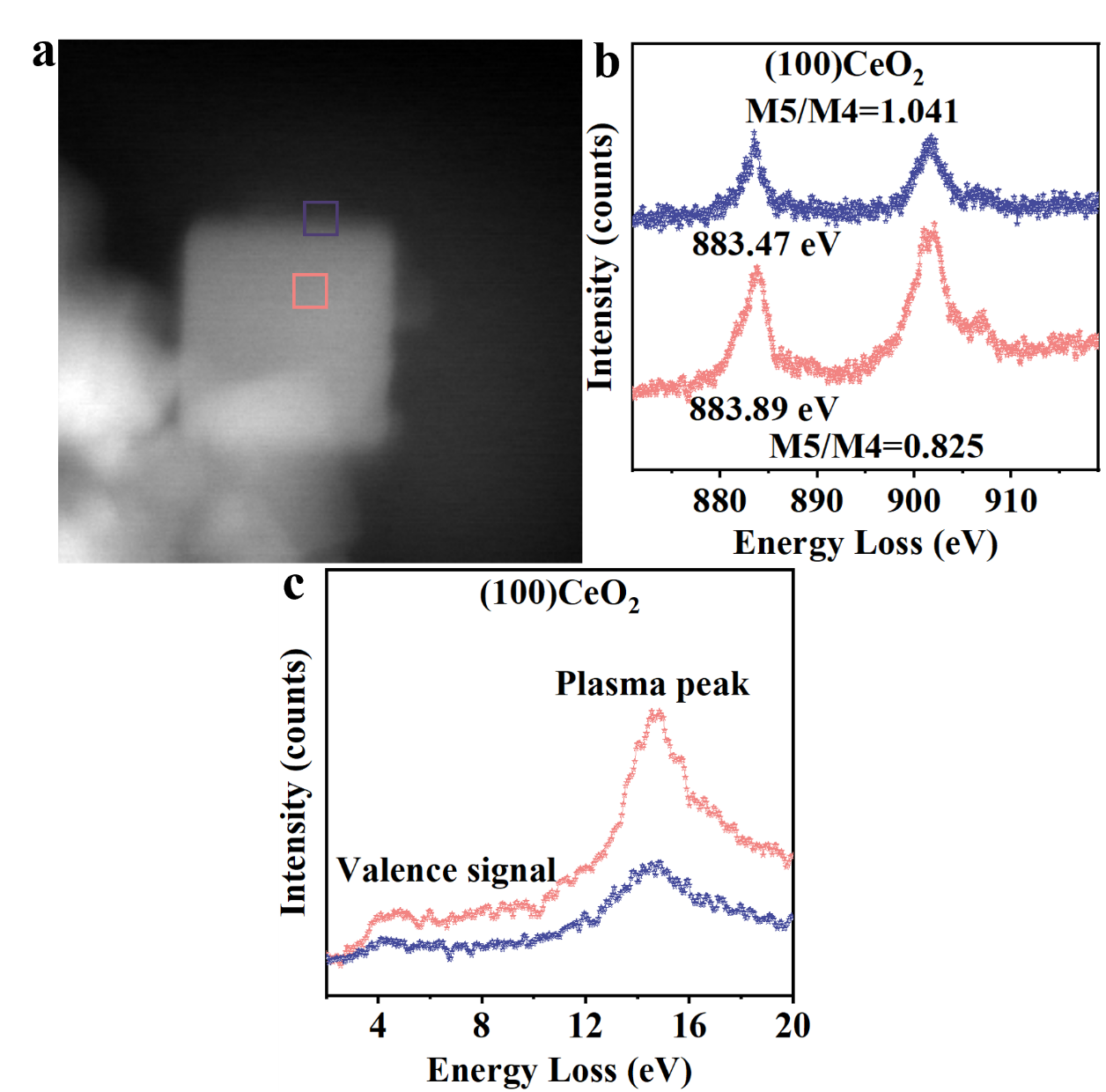


**Figure S5.** HAADF images of (100)CeO_2_ and corresponding valence electron energy loss spectra.


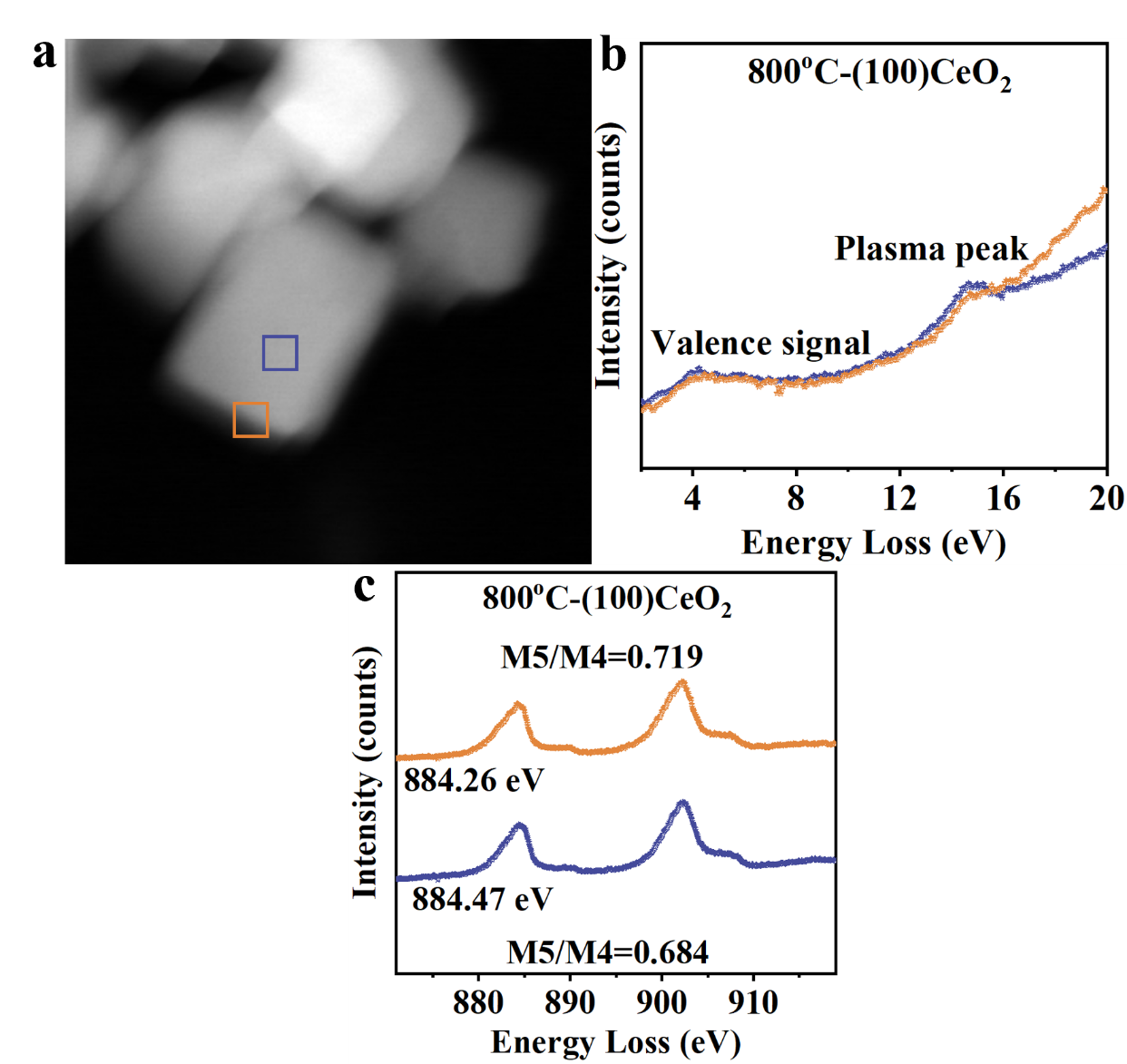


**Figure S6.** HAADF images of 800^o^C-(100)CeO_2_ and corresponding valence electron energy loss spectra.


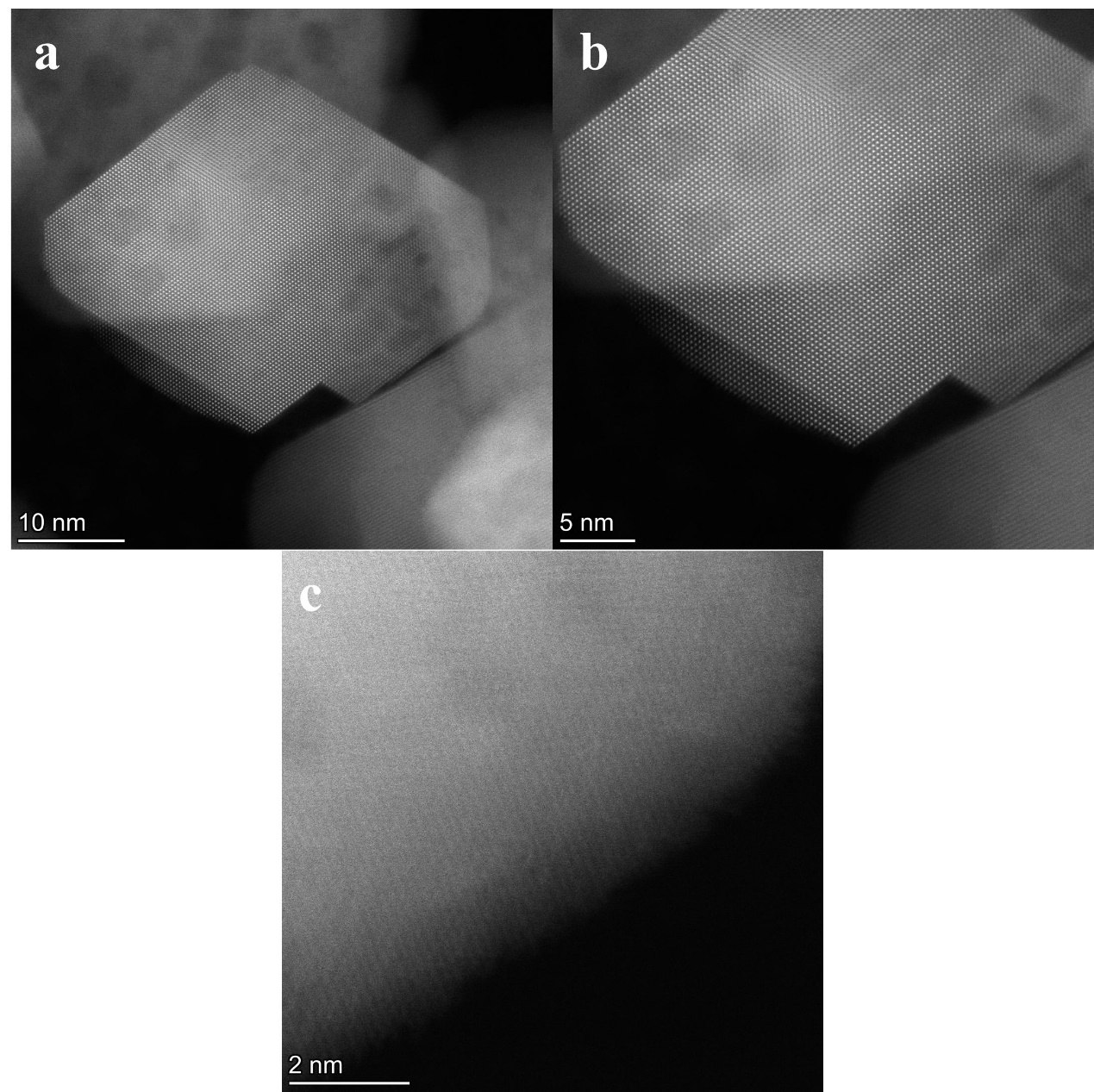


**Figure S7.** HAADF images of Pt_SA_-(111)CeO_2_.


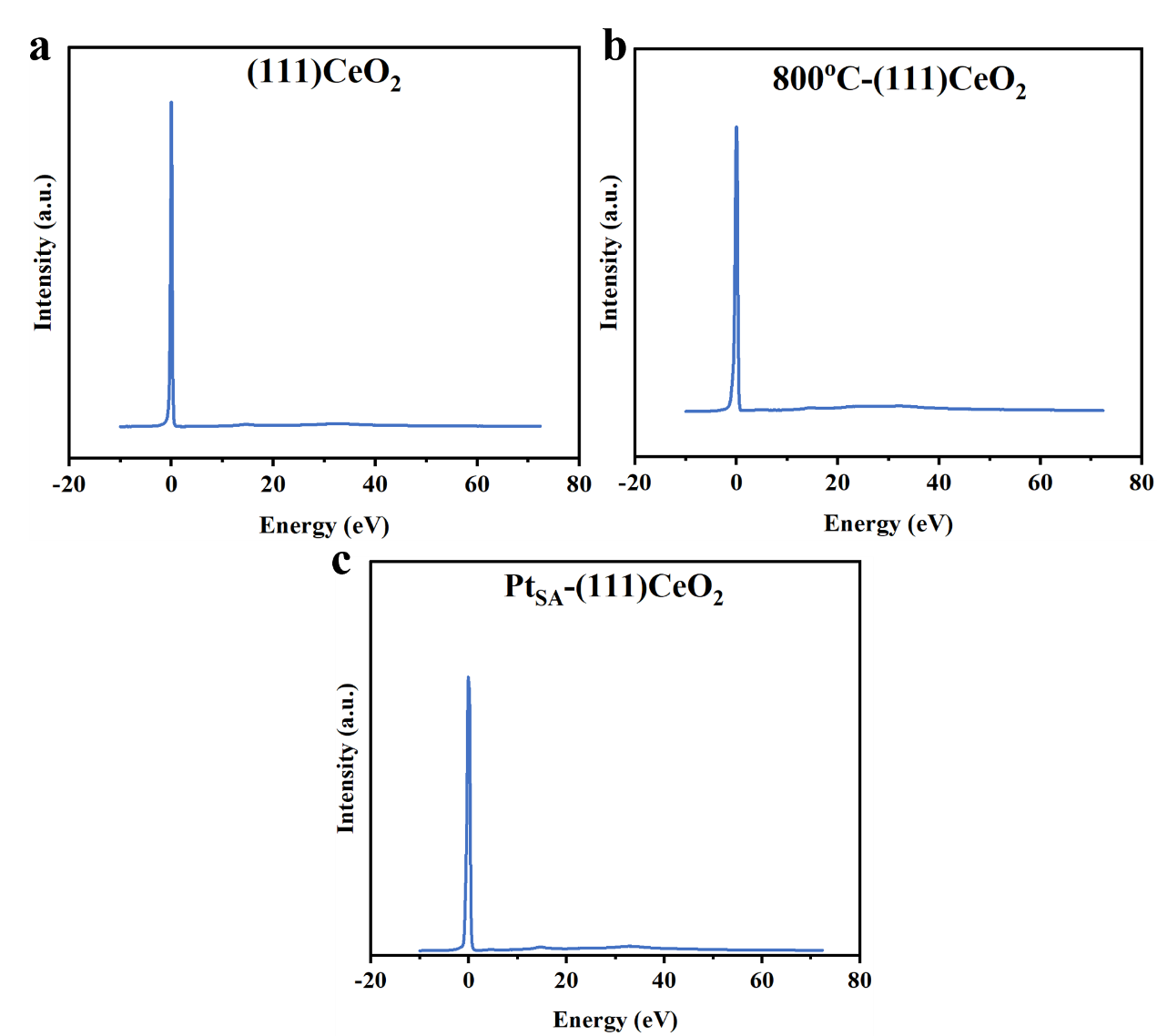


**Figure S8.** Zero-loss peak of (111)CeO_2_,800^o^C-(111)CeO_2_ and

Pt_SA_-(111)CeO_2_.


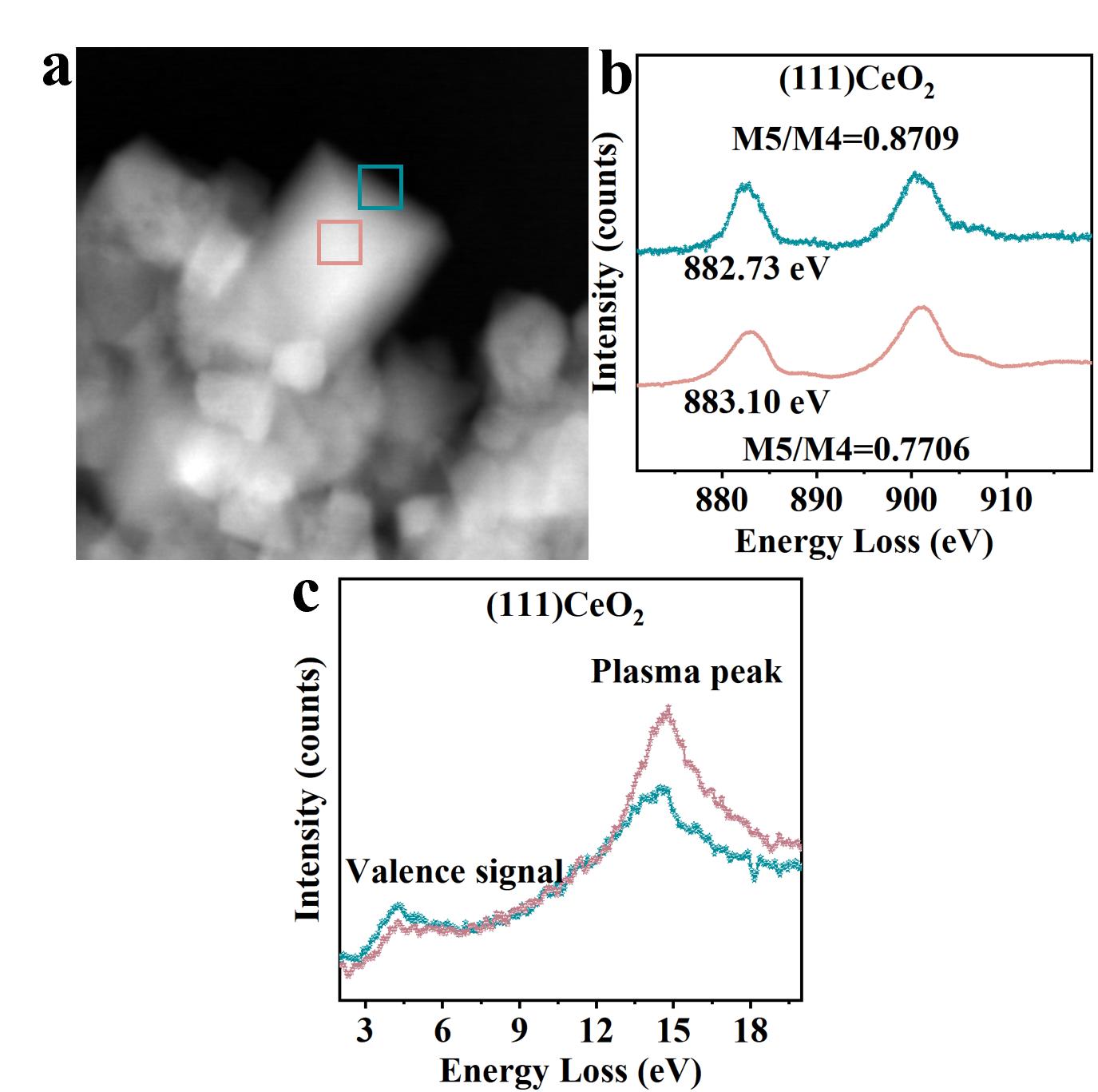


**Figure S9.** HAADF images of (111)CeO_2_ and corresponding valence electron energy loss spectra.


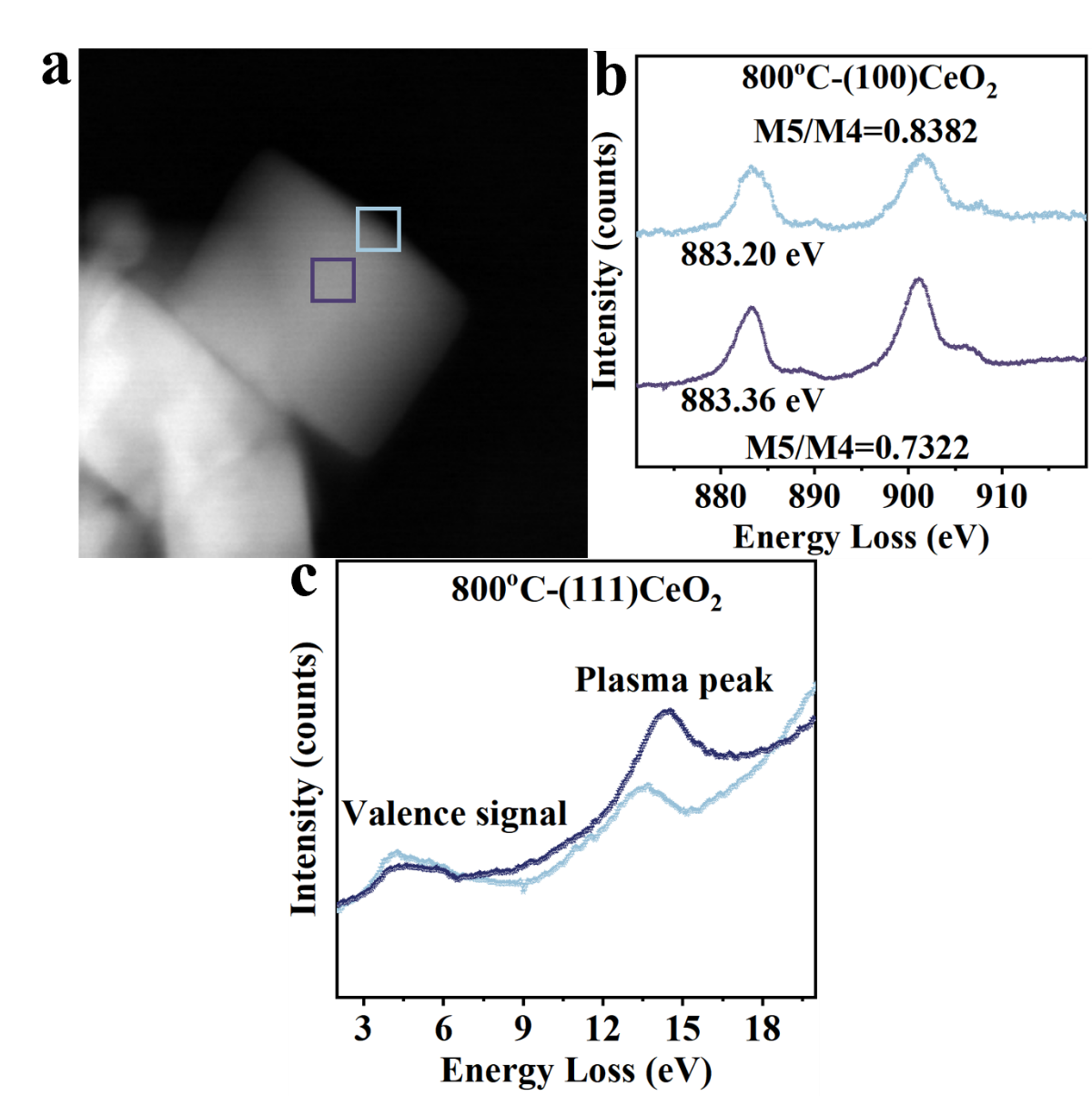


**Figure S10.** HAADF images of 800^o^C-(111)CeO_2_ and corresponding valence electron energy loss spectra.


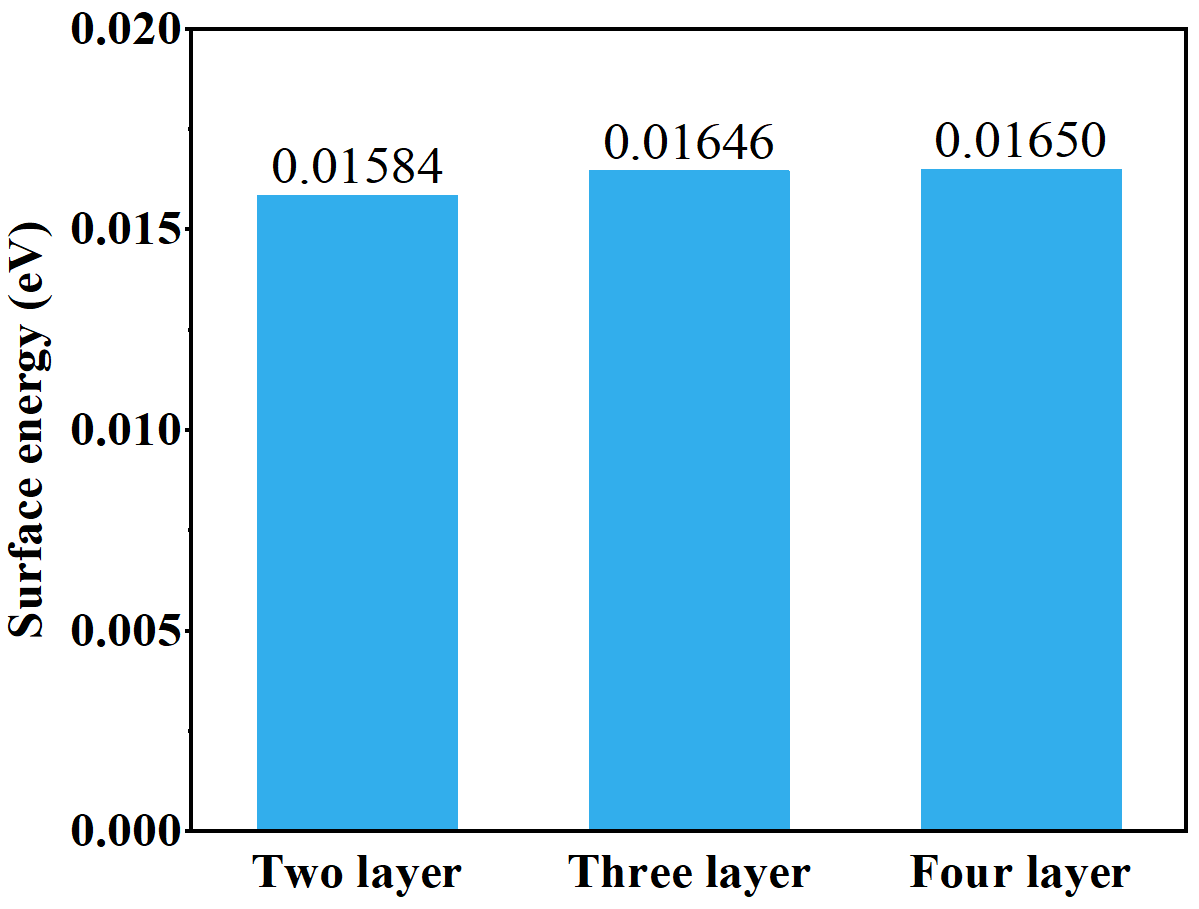


**Figure S11.** The surface energies of (100)CeO_2_ with various layers.


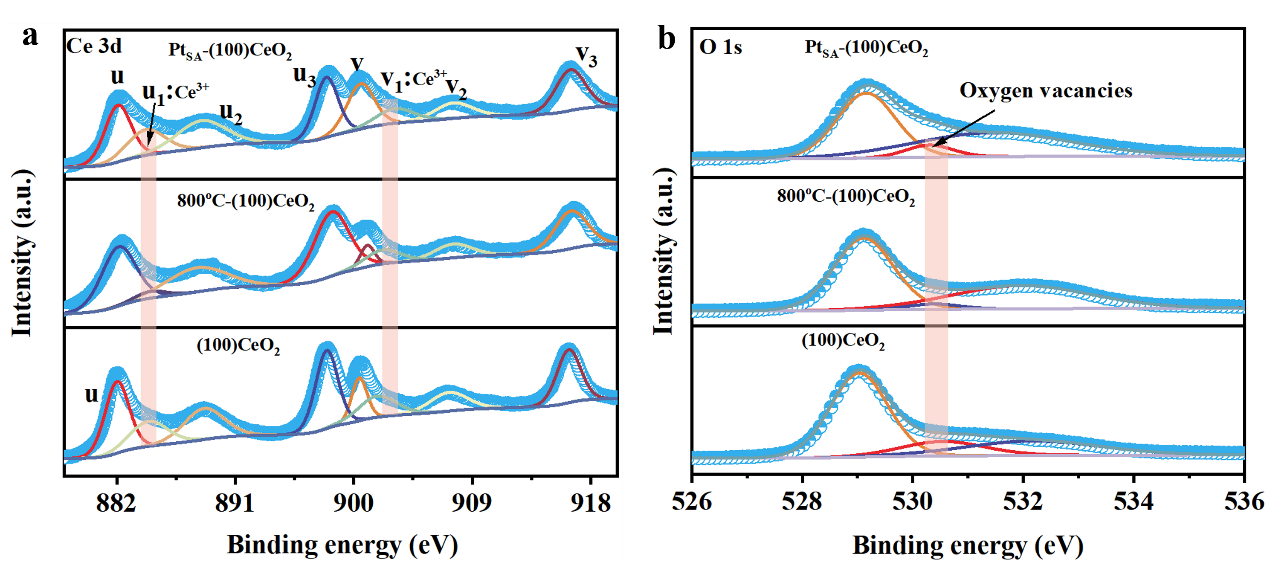


**Figure S12.** Ce 3d and O 1s high-resolution XPS spectra of (100)CeO_2_, 800^o^C-(100)CeO_2_ and Pt_SA_-(100)CeO_2_.


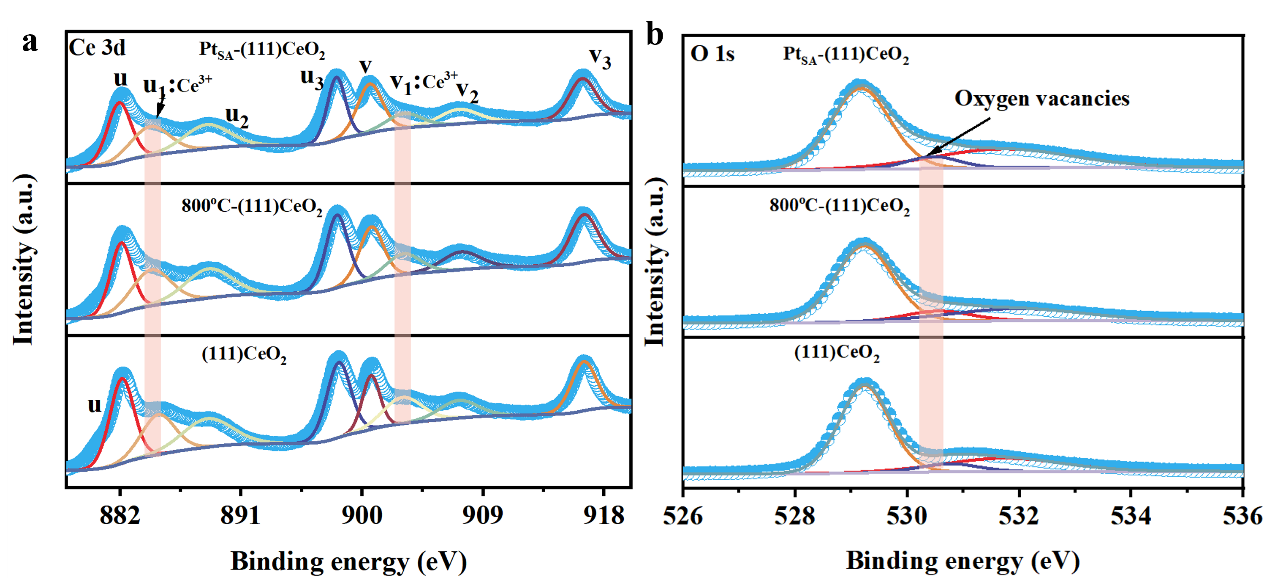


**Figure S13.** Ce 3d and O 1s high-resolution XPS spectra of (111)CeO_2_, 800^o^C-(111)CeO_2_ and Pt_SA_-(111)CeO_2_.


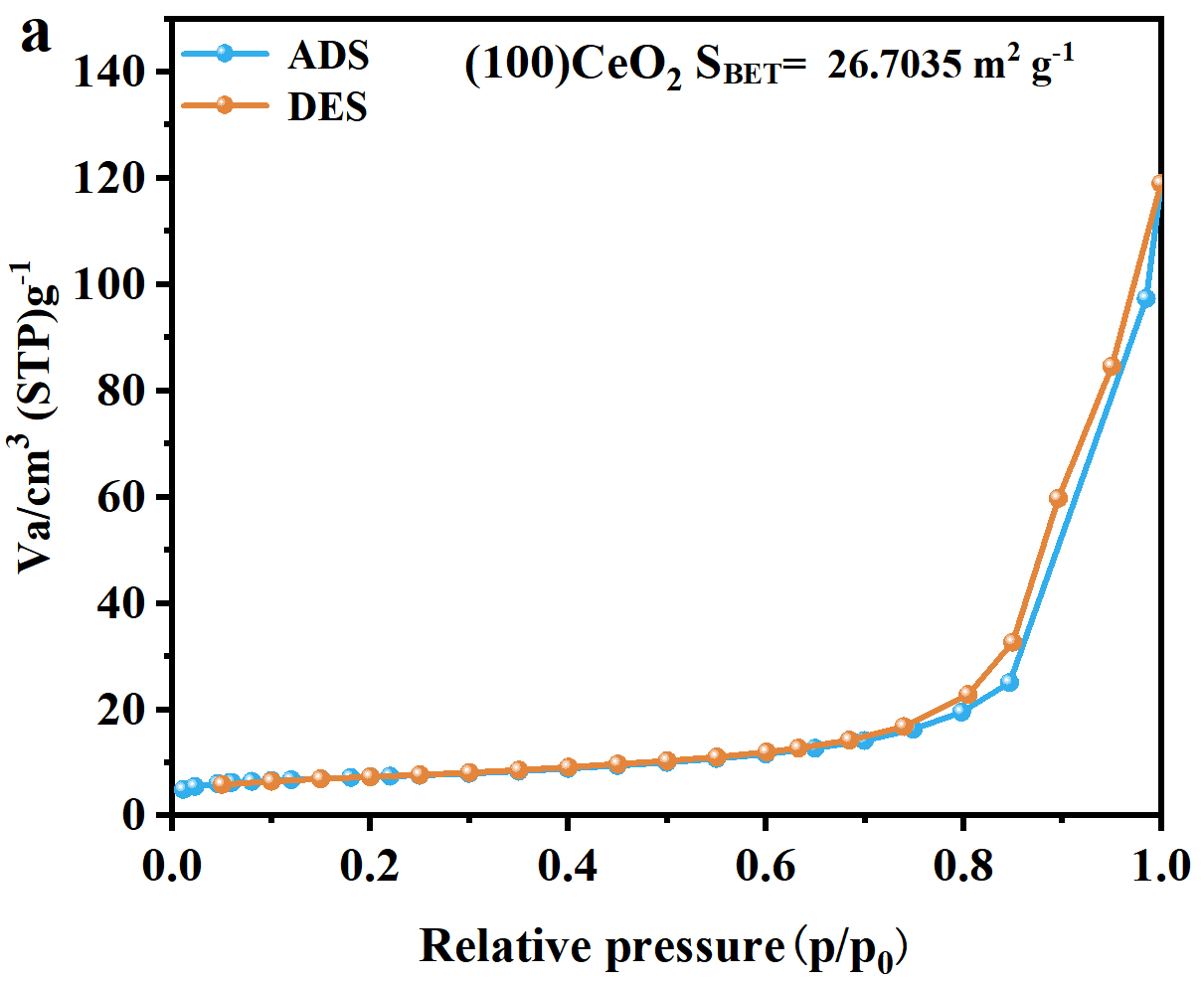

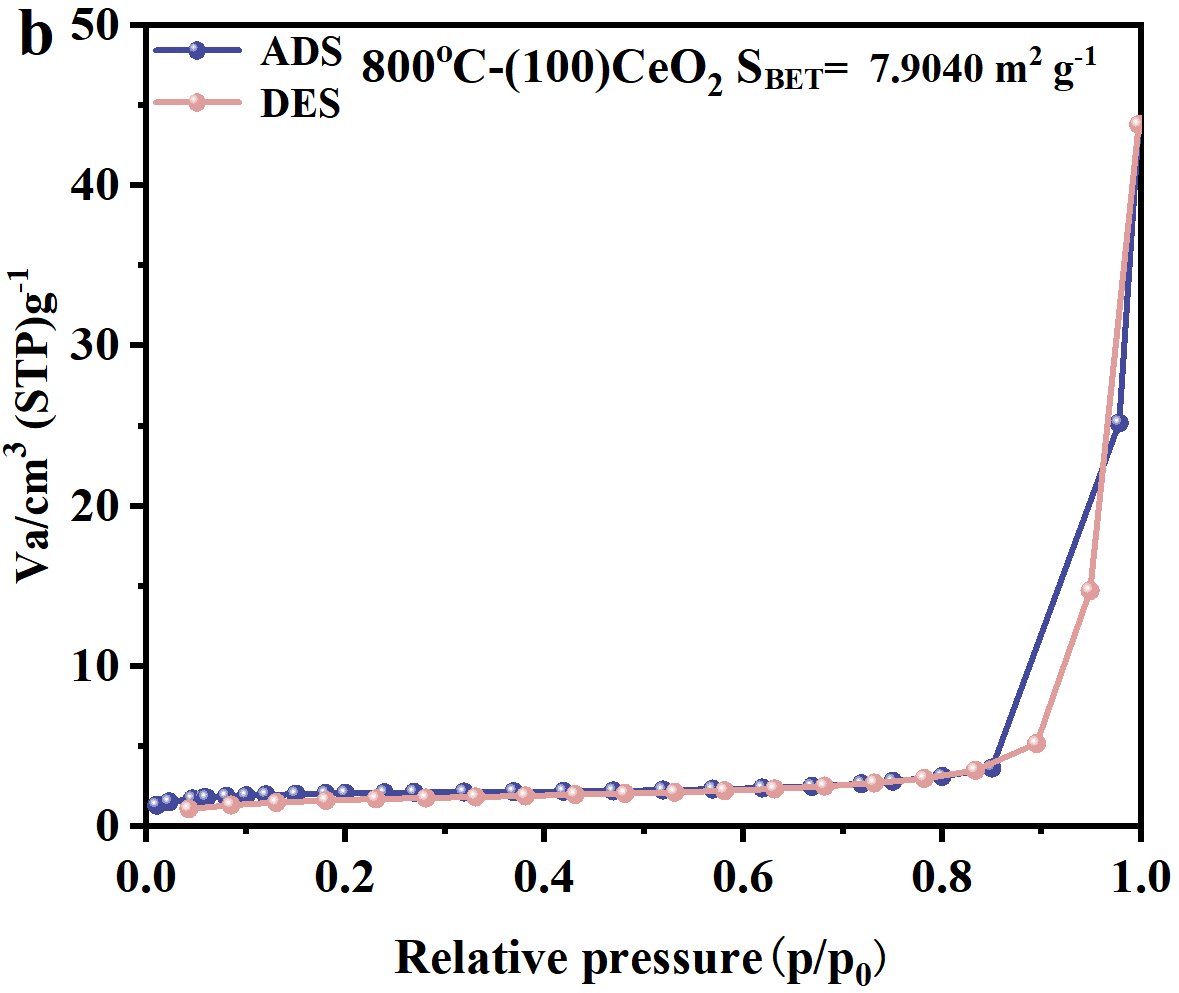


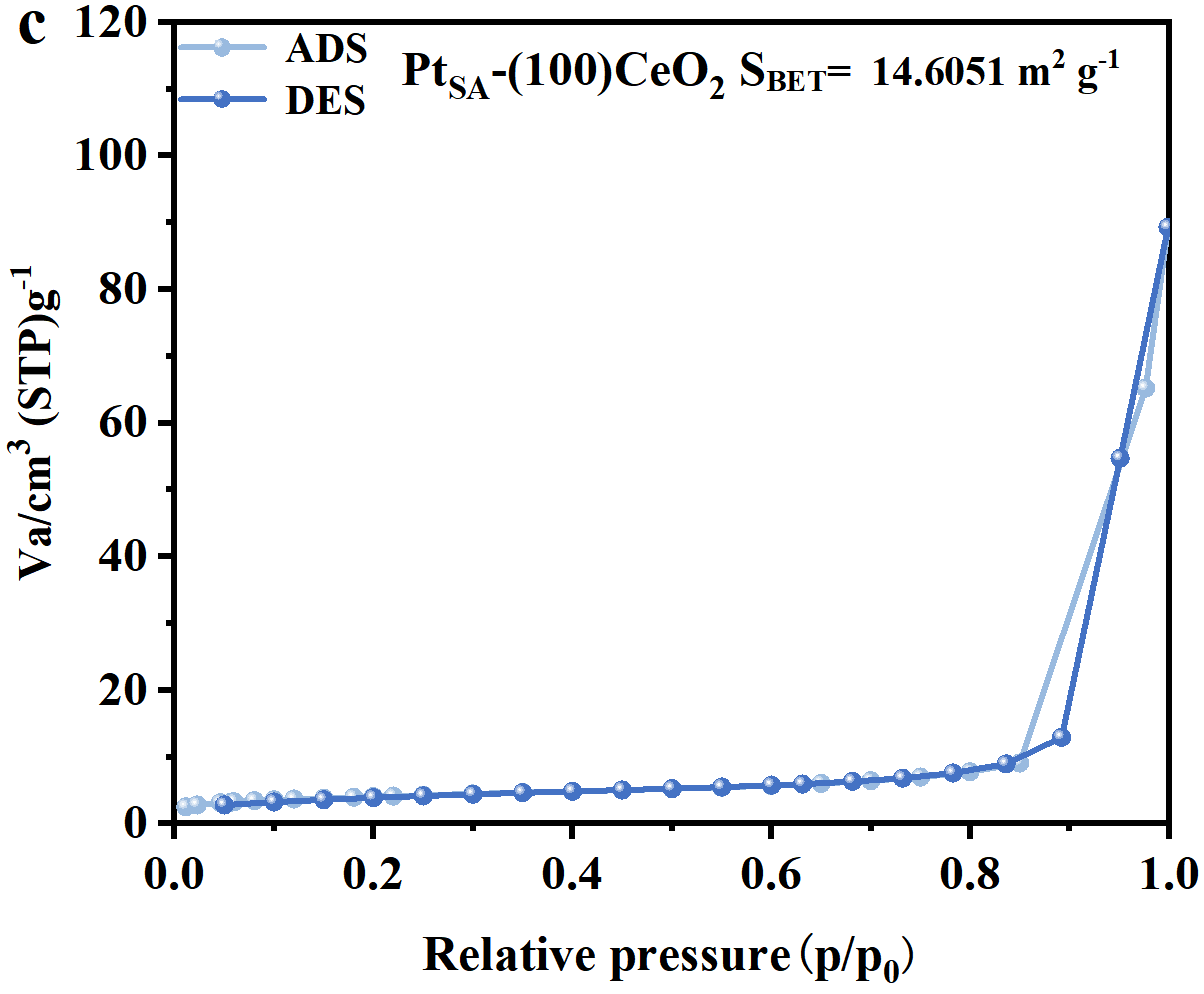


**Figure S14.** N2 adsorption-desorption isotherms of (100)CeO_2_, 800^o^C-(100)CeO_2_ and Pt_SA_-(100)CeO_2_.


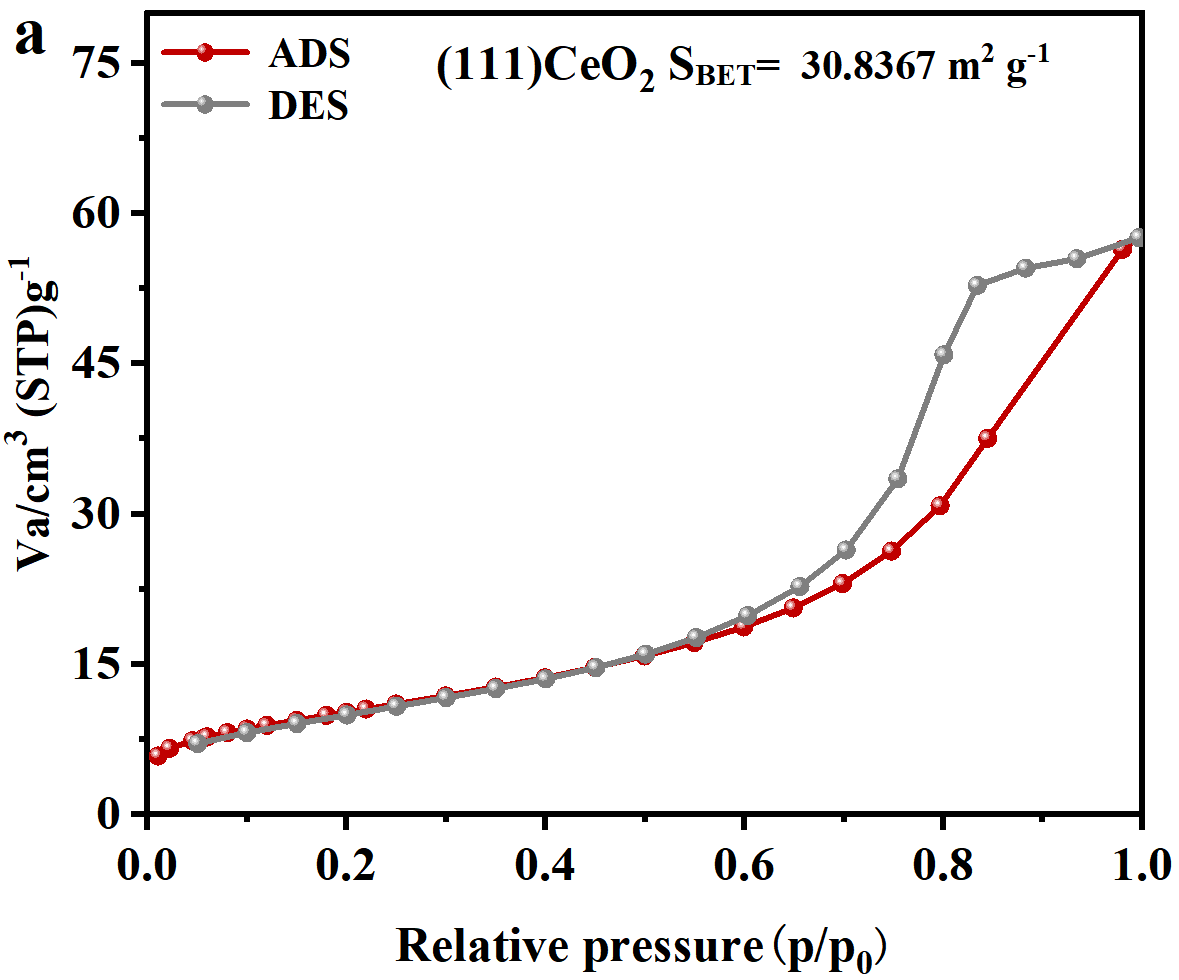

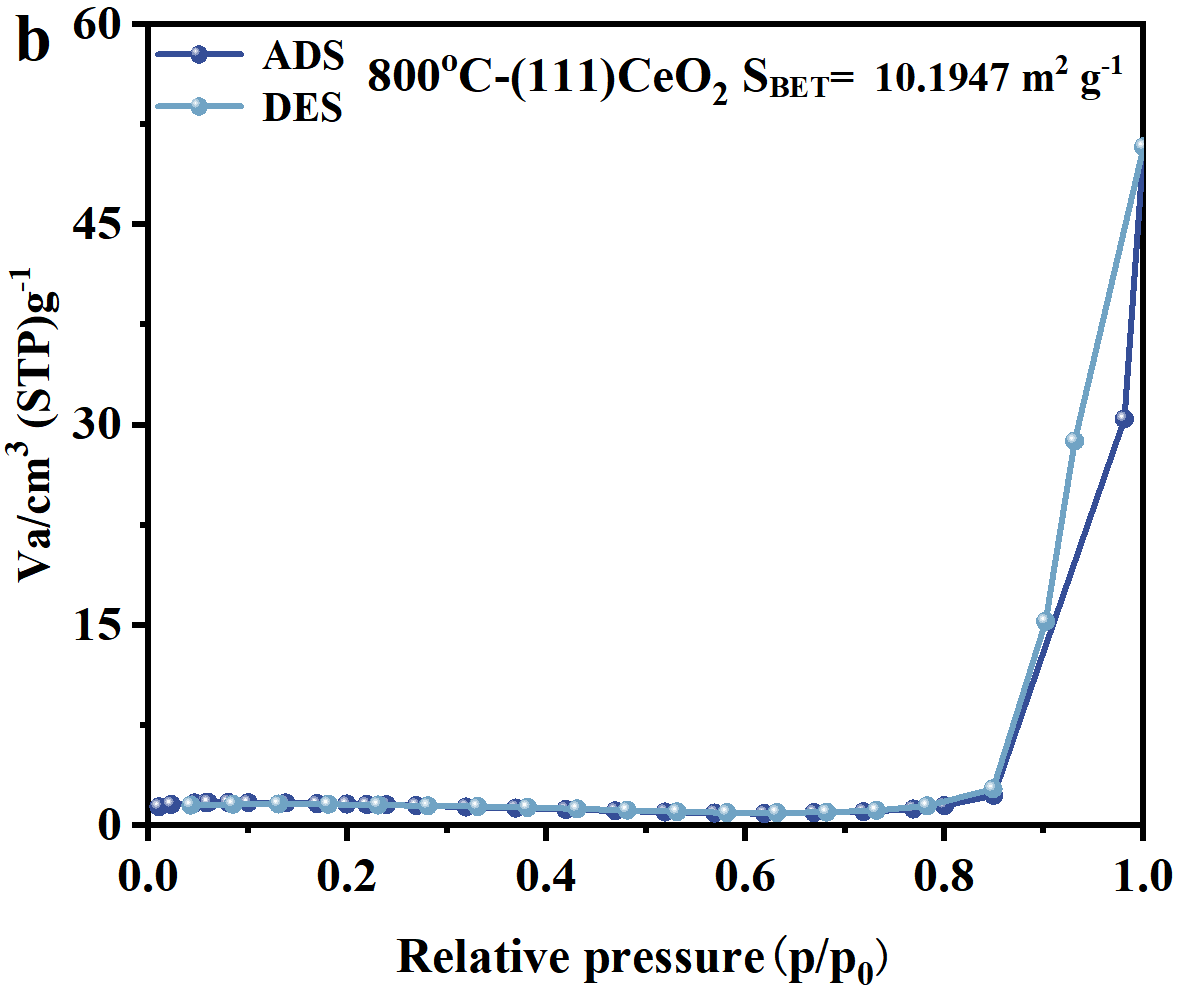

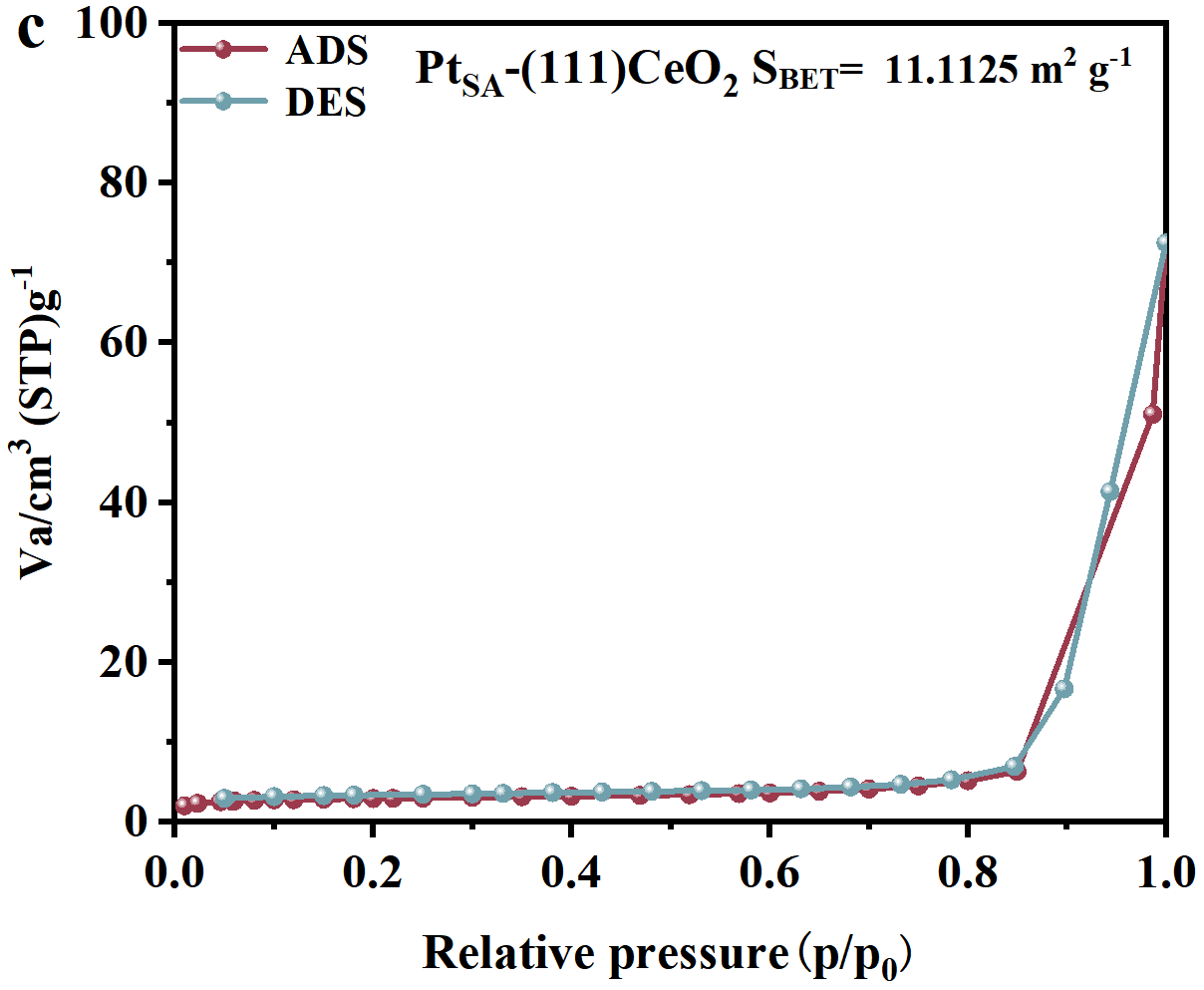


**Figure S15.** N2 adsorption-desorption isotherms of (111)CeO_2_, 800^o^C-(111)CeO_2_ and Pt_SA_-(111)CeO_2_.


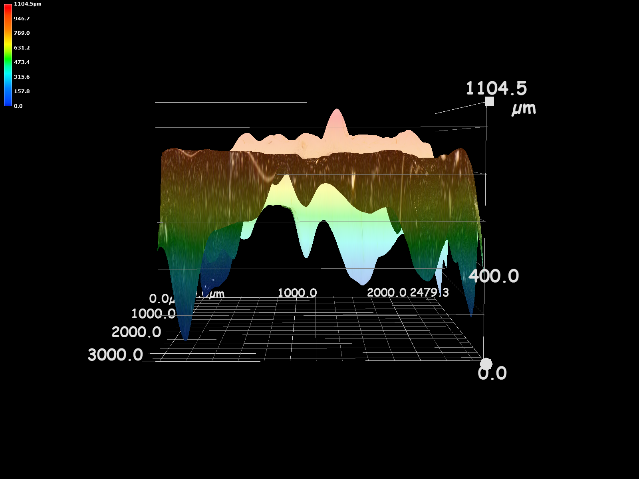


**Figure S16.** The thickness of sensing layer.


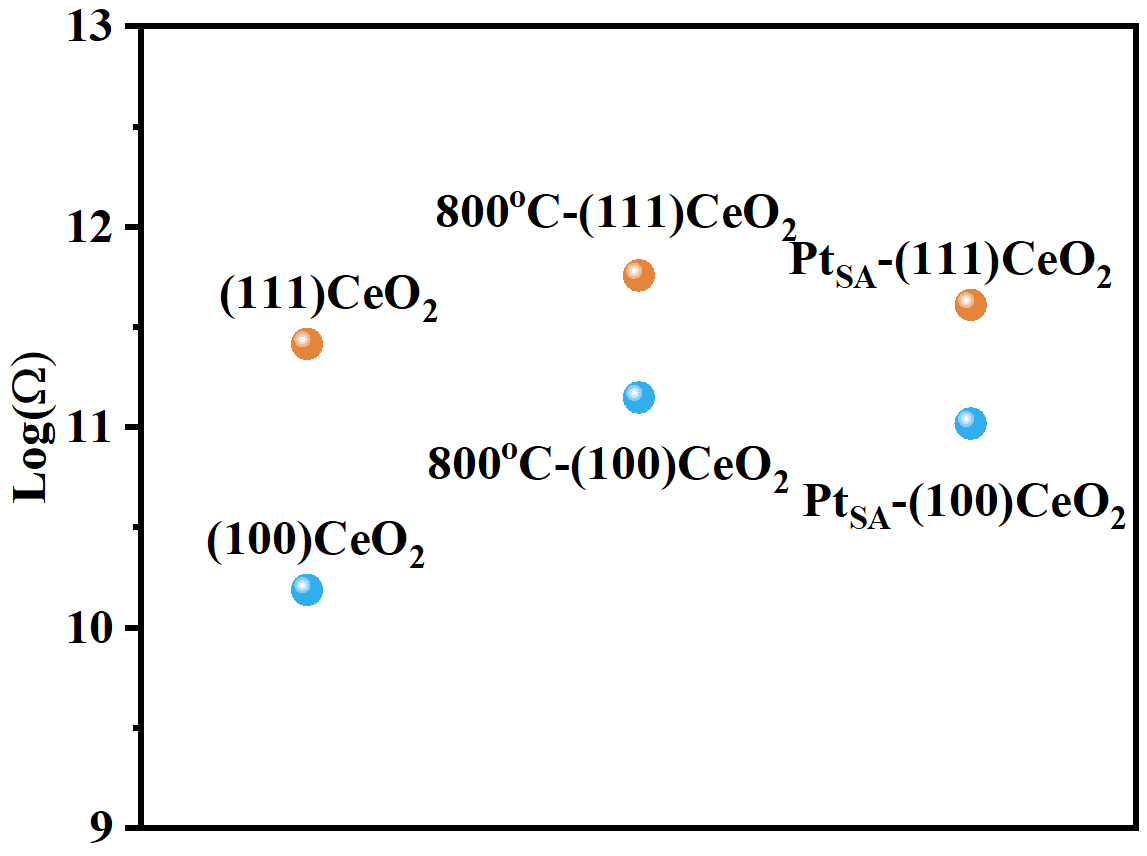


**Figure S17.** The resistance of (111)CeO_2_, 800^o^C-(111)CeO_2_, Pt_SA_-(111)CeO_2_ and (100)CeO_2_, 800^o^C-(100)CeO_2_, Pt_SA_-(100)CeO_2_.


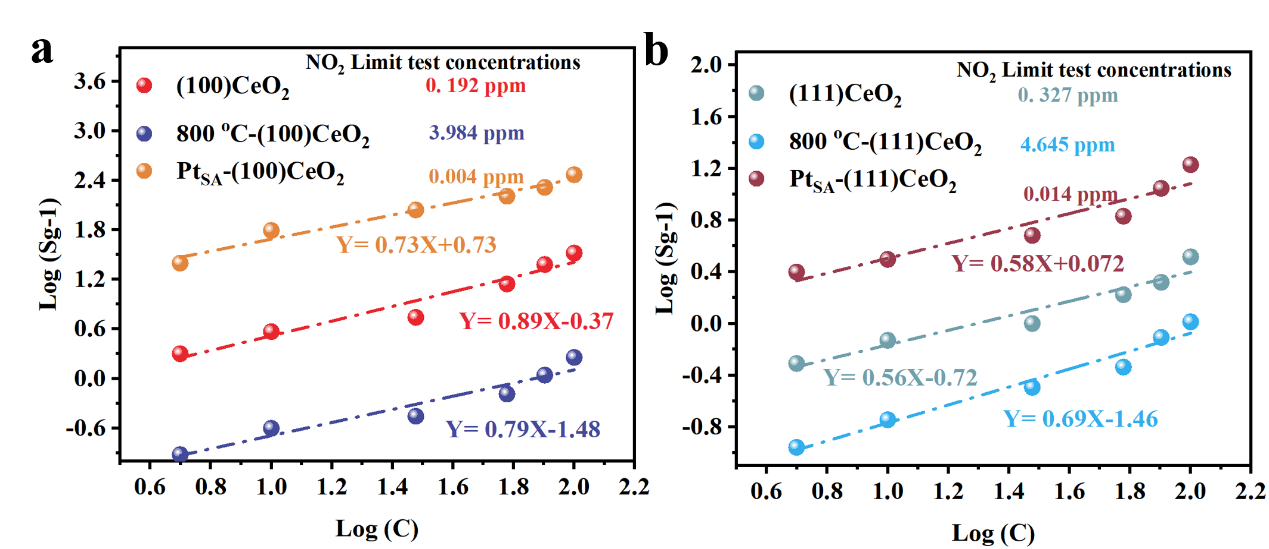


**Figure S18.** corresponding log(Sg-1) versus log(Cg) curves,.


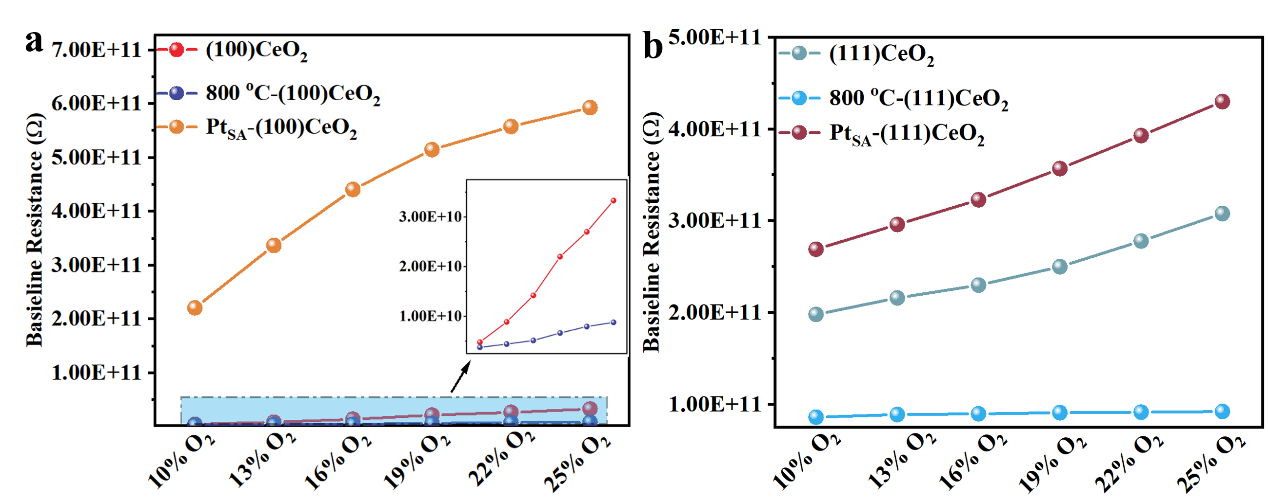


**Figure S19.** The resistance of various sample under different concentration of O_2_.


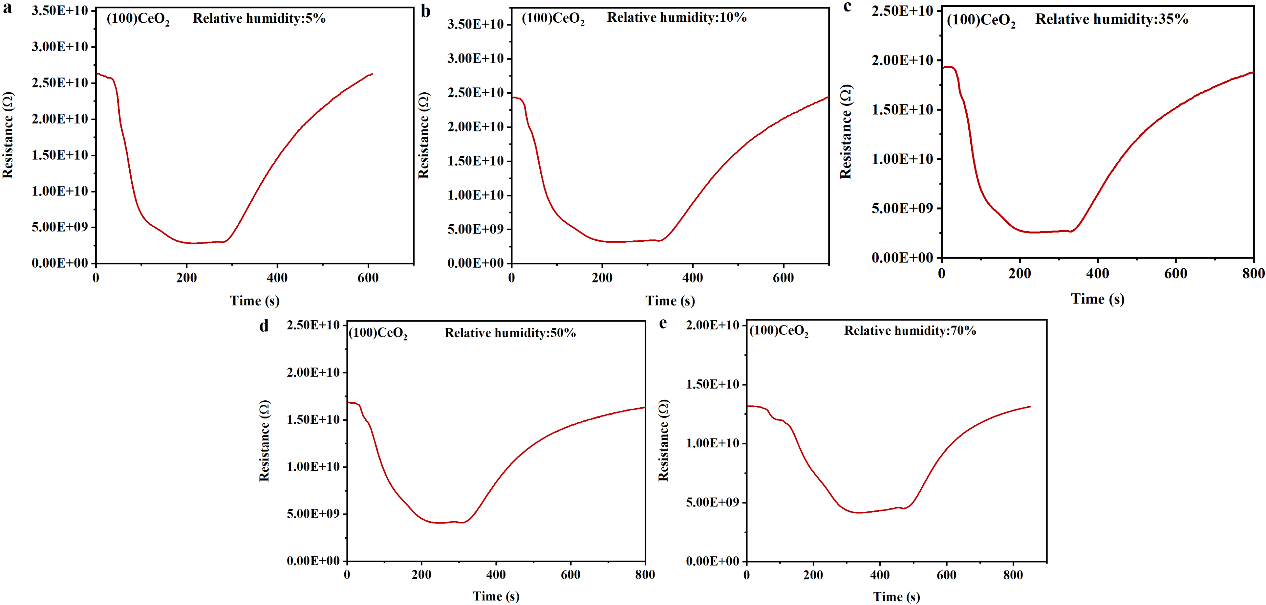


**Figure S20.** The gas performance of (100)CeO_2_ toward 30 ppm NO_2_ under different relative humidity.


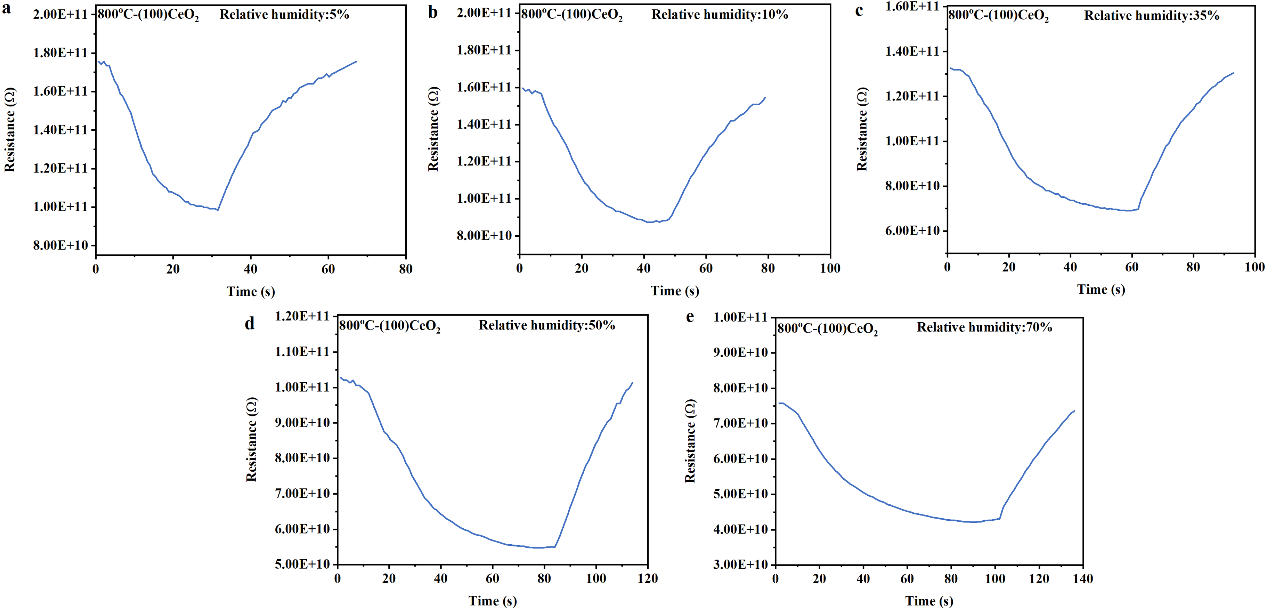


**Figure S21.** The gas performance of 800^o^C-(100)CeO_2_ toward 30 ppm NO_2_ under different relative humidity.


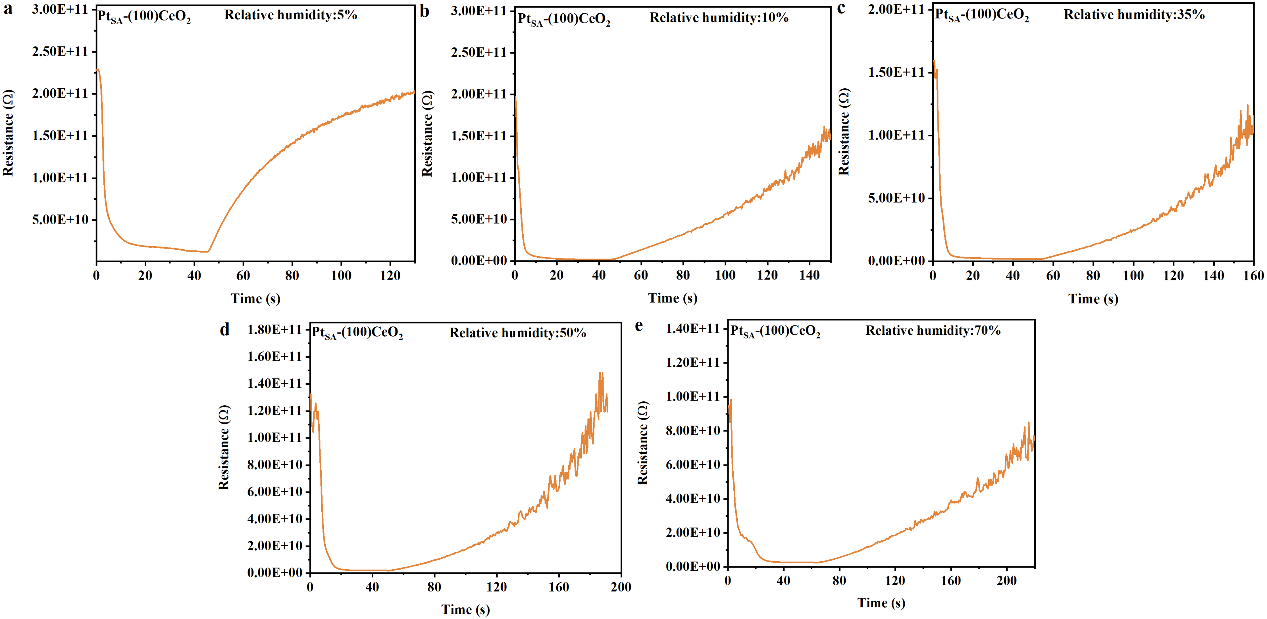


**Figure S22.** The gas performance of Pt_SA_-(100)CeO_2_ toward 30 ppm NO_2_ under different relative humidity.


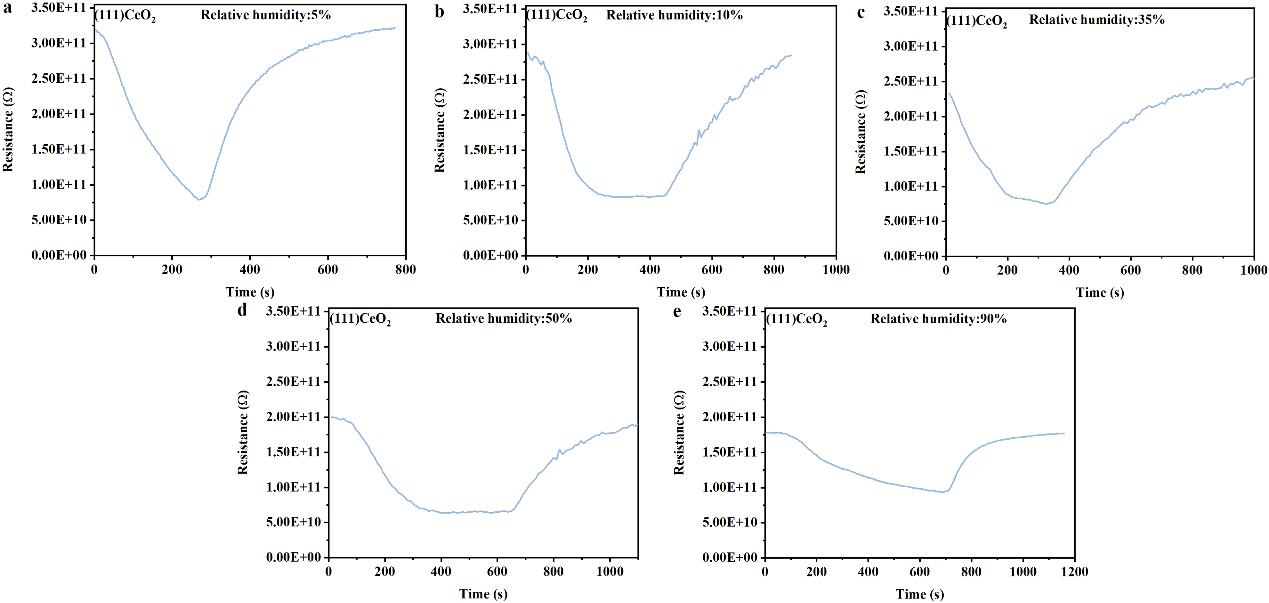


**Figure S23.** The gas performance of (111)CeO_2_ toward 30 ppm NO_2_ under different relative humidity.


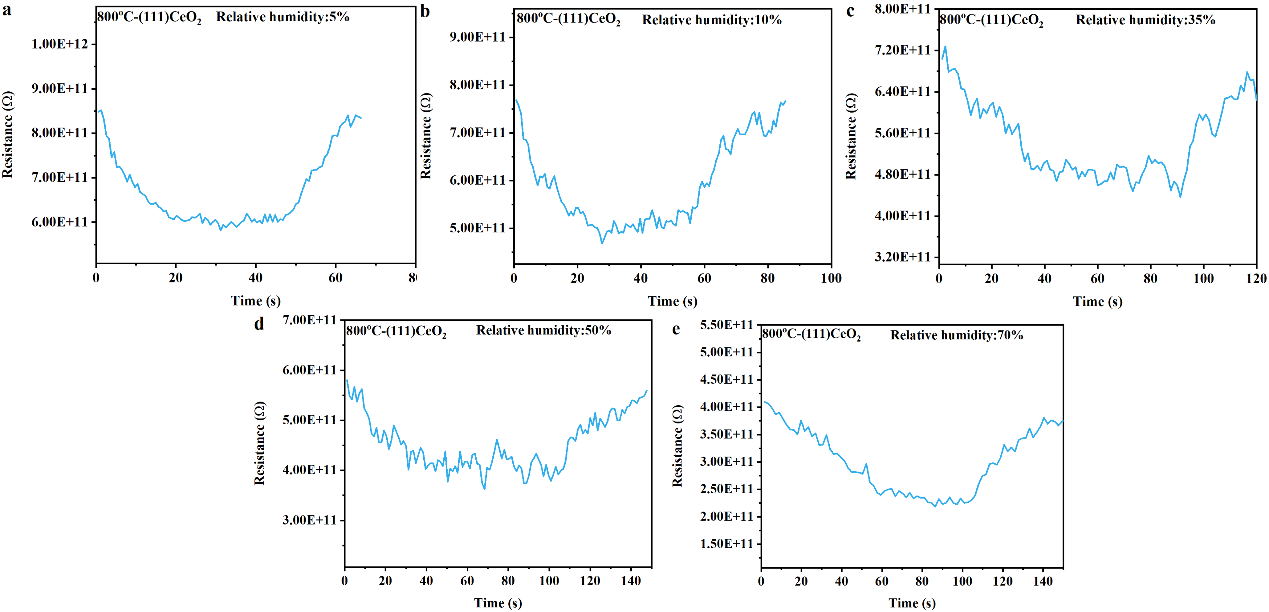


**Figure S24.** The gas performance of 800^o^C-(111)CeO_2_ toward 30 ppm NO_2_ under different relative humidity.


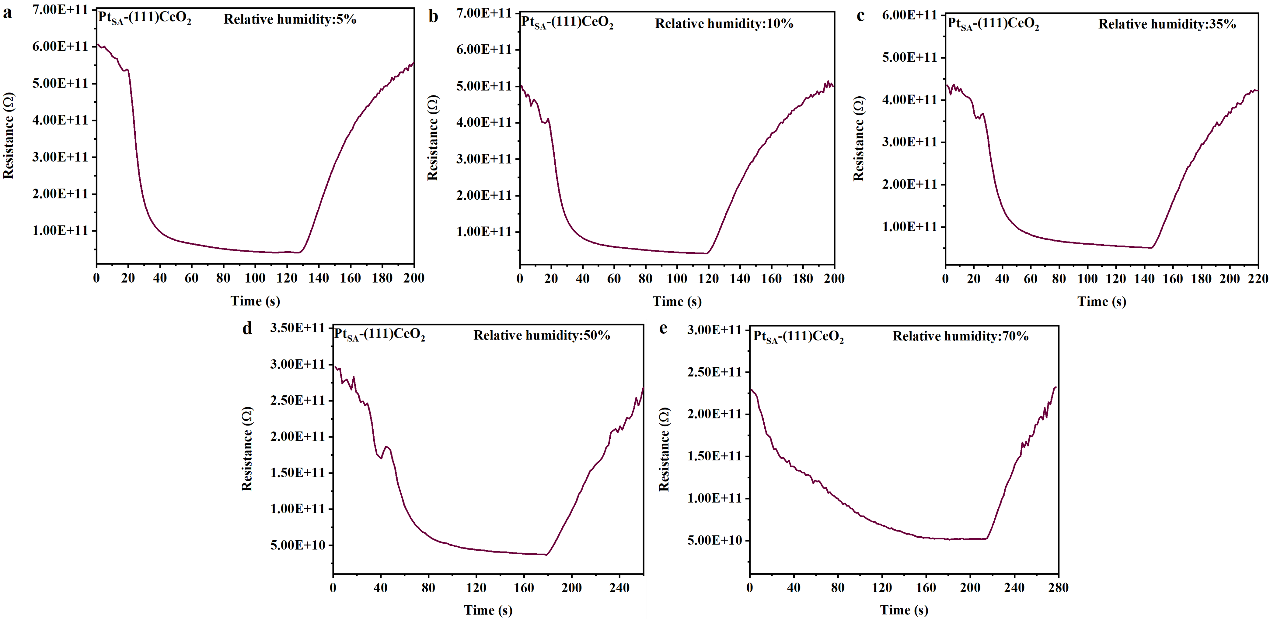


**Figure S25.** The gas performance of Pt_SA_-(111)CeO_2_ toward 30 ppm NO_2_ under different relative humidity.


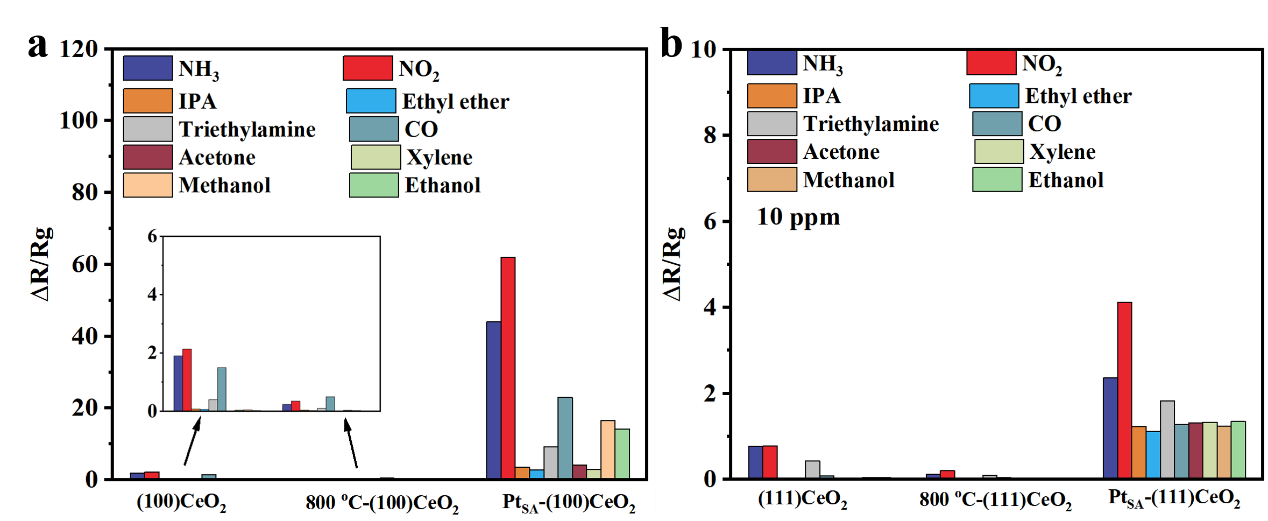


**Figure S26.** The selectivity of various samples toward different target molecules.


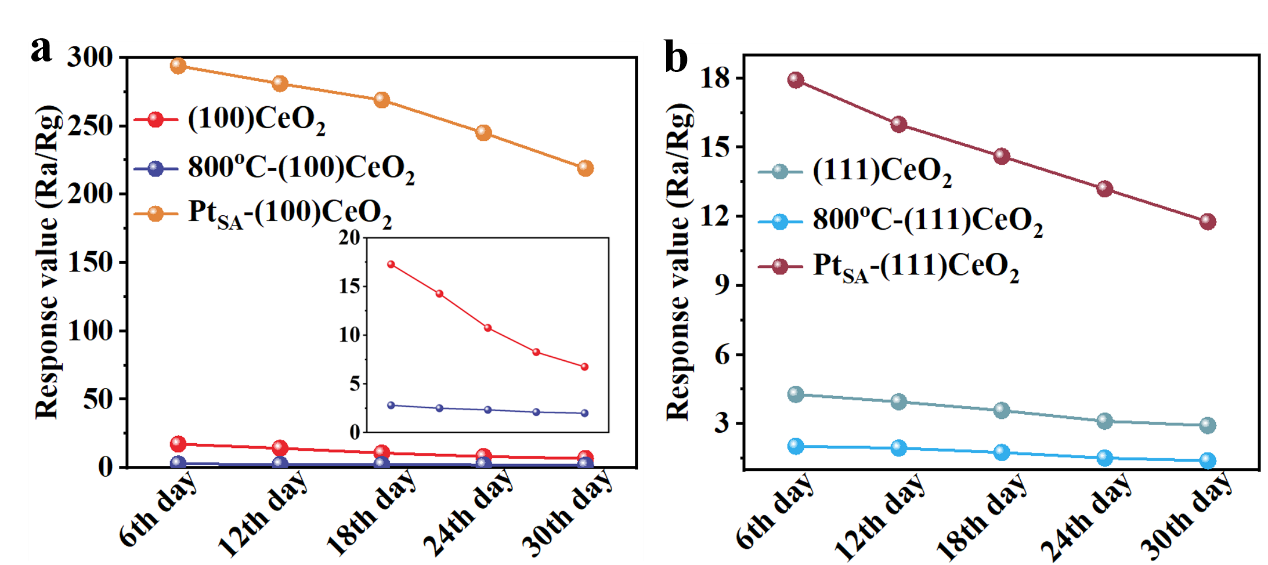


**Figure S27.** The long-term stability of various samples.


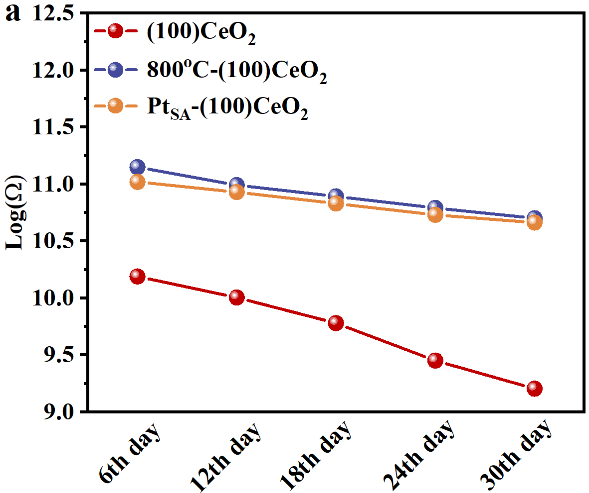

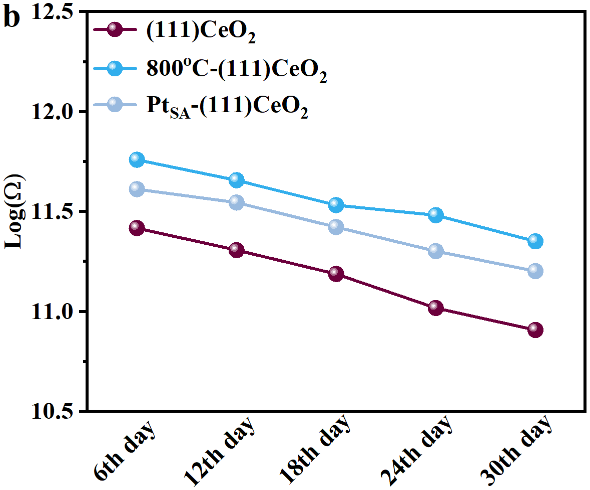


**Figure S28.** The baseline resistance of various samples under long-term stability test.


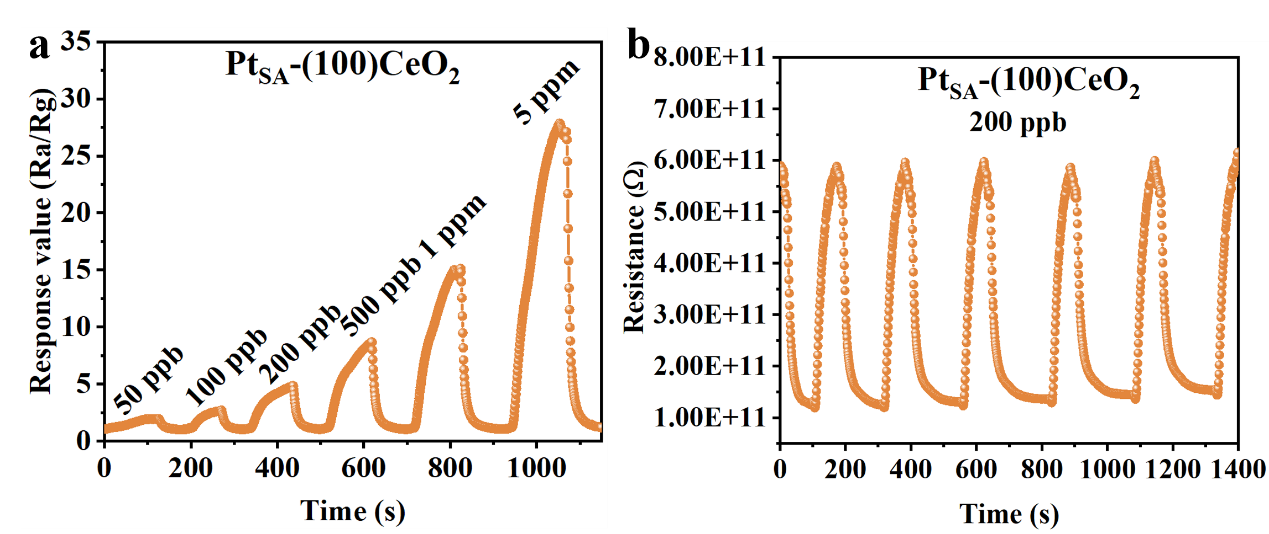
**Figure S29.** The dynamic sensing transient curves for different concentrations of NO_2_ of Pt_SA_-(100)CeO_2_.


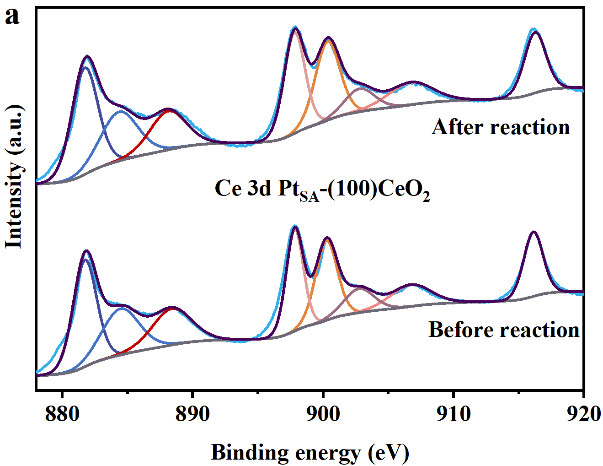

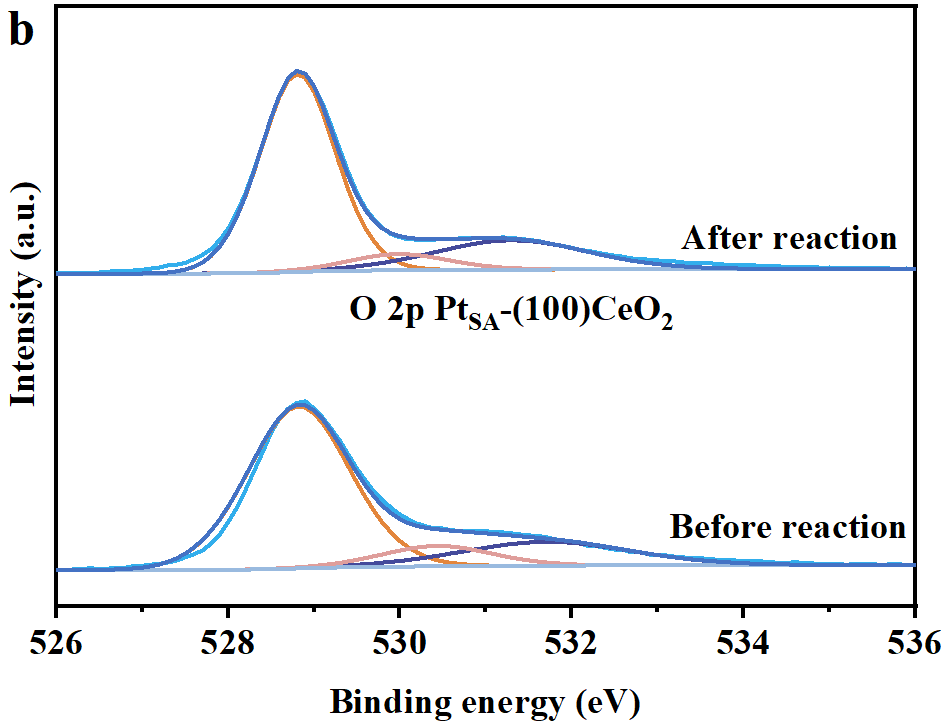


**Figure. S30.** a) Ce 3d high-resolution XPS spectra of Pt_SA_-(100)CeO_2_ after reaction, b) O 2p high-resolution XPS spectra of Pt_SA_-(100)CeO_2_ after reaction.


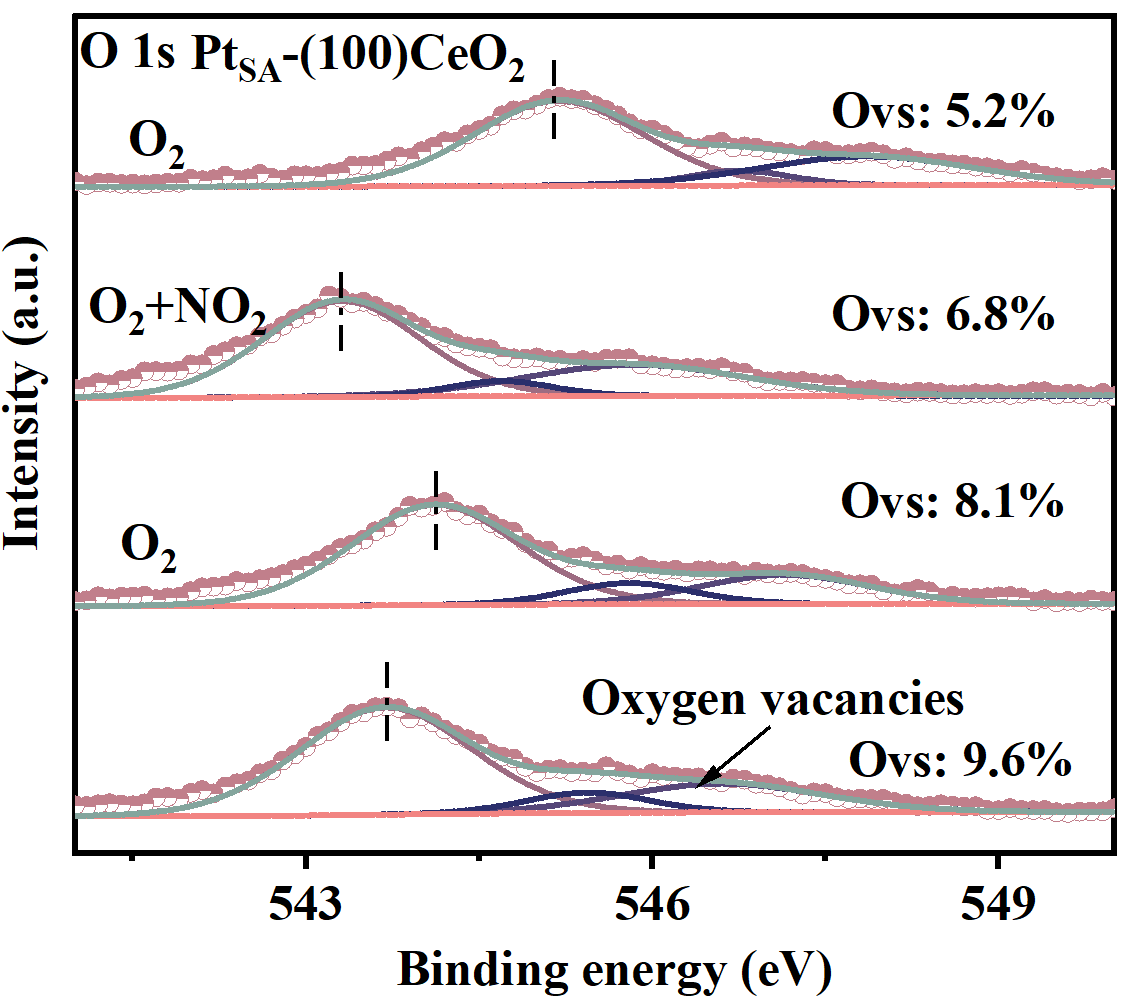


**Figure S31.** In situ NAP-XPS O 1s of Pt_SA_-(100)CeO_2_ recorded at room temperature after exposed to O_2_, NO_2_+O_2_ and O_2_.


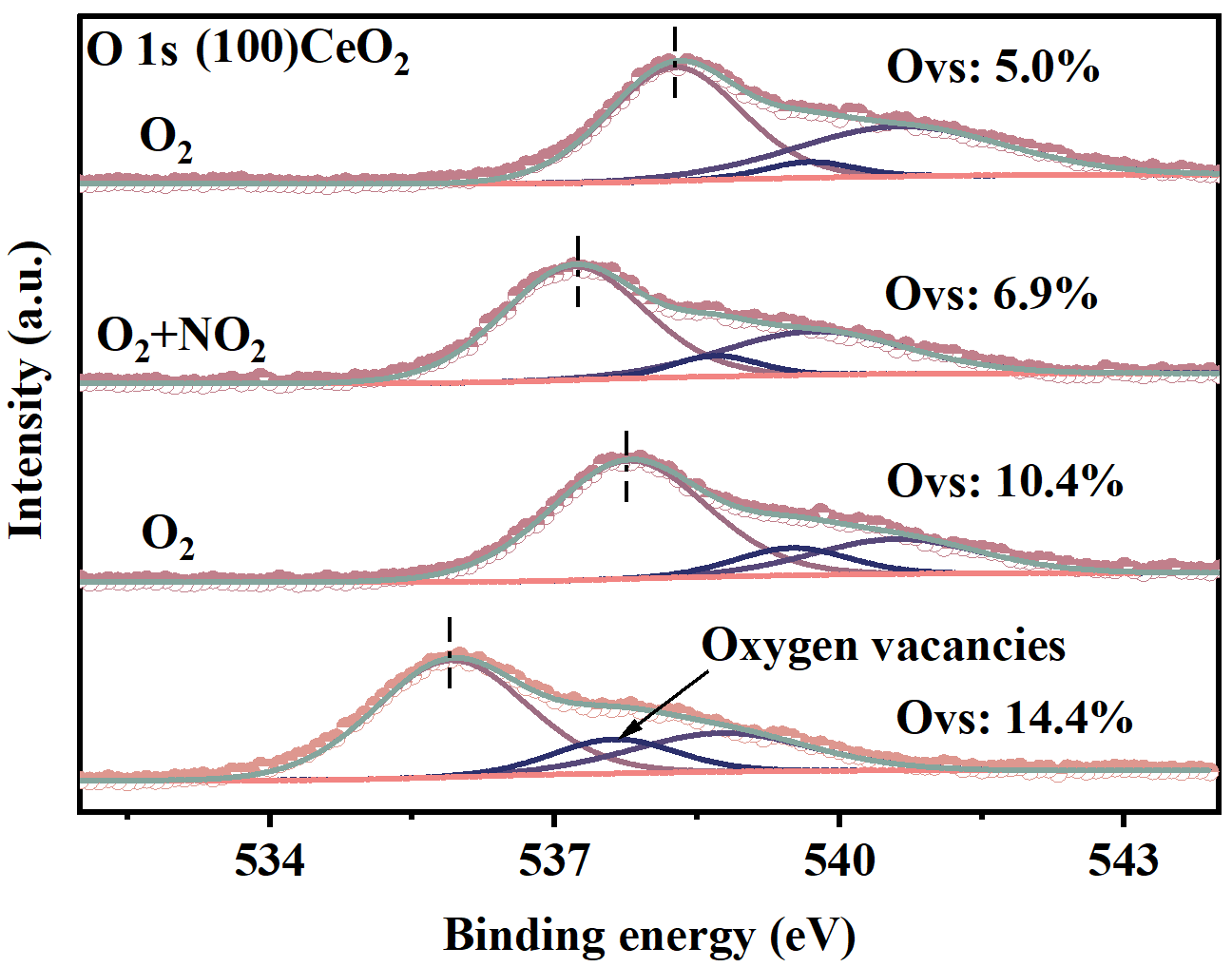


**Figure S32.** In situ NAP-XPS O 1s of (100)CeO_2_ recorded at room temperature after exposed to O_2_, NO_2_+O_2_ and O_2_.


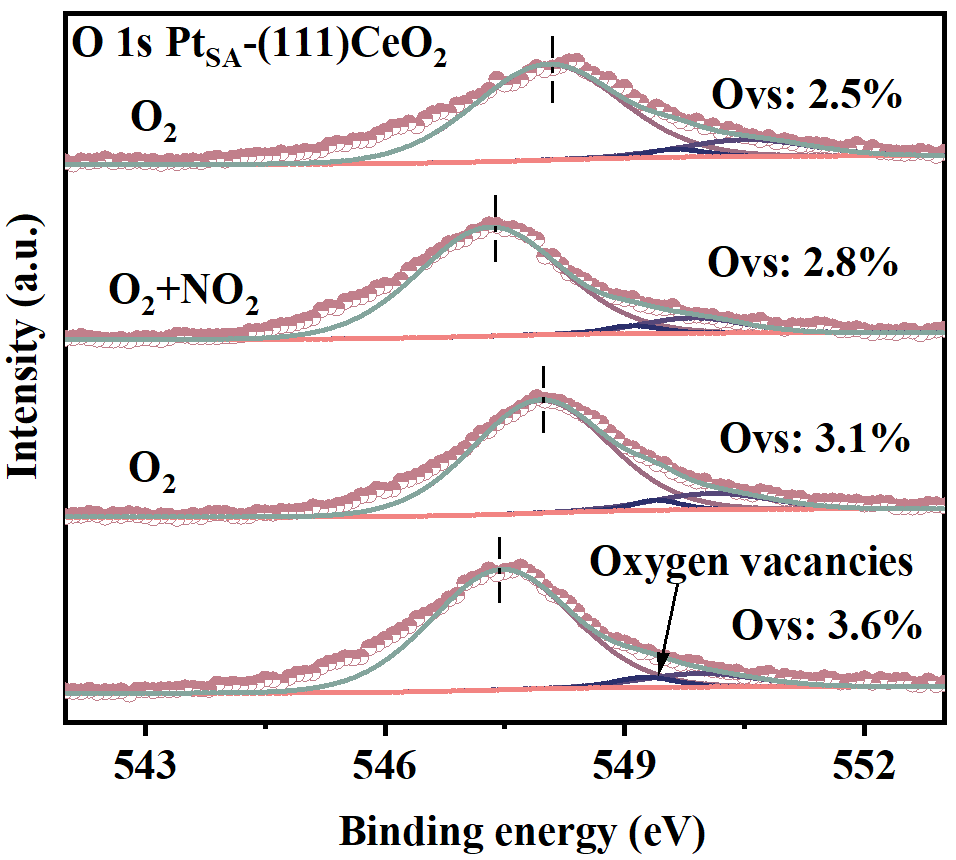


**Figure S33.** In situ NAP-XPS O 1s of Pt_SA_-(111)CeO_2_ recorded at room temperature after exposed to O_2_, NO_2_+O_2_ and O_2_.


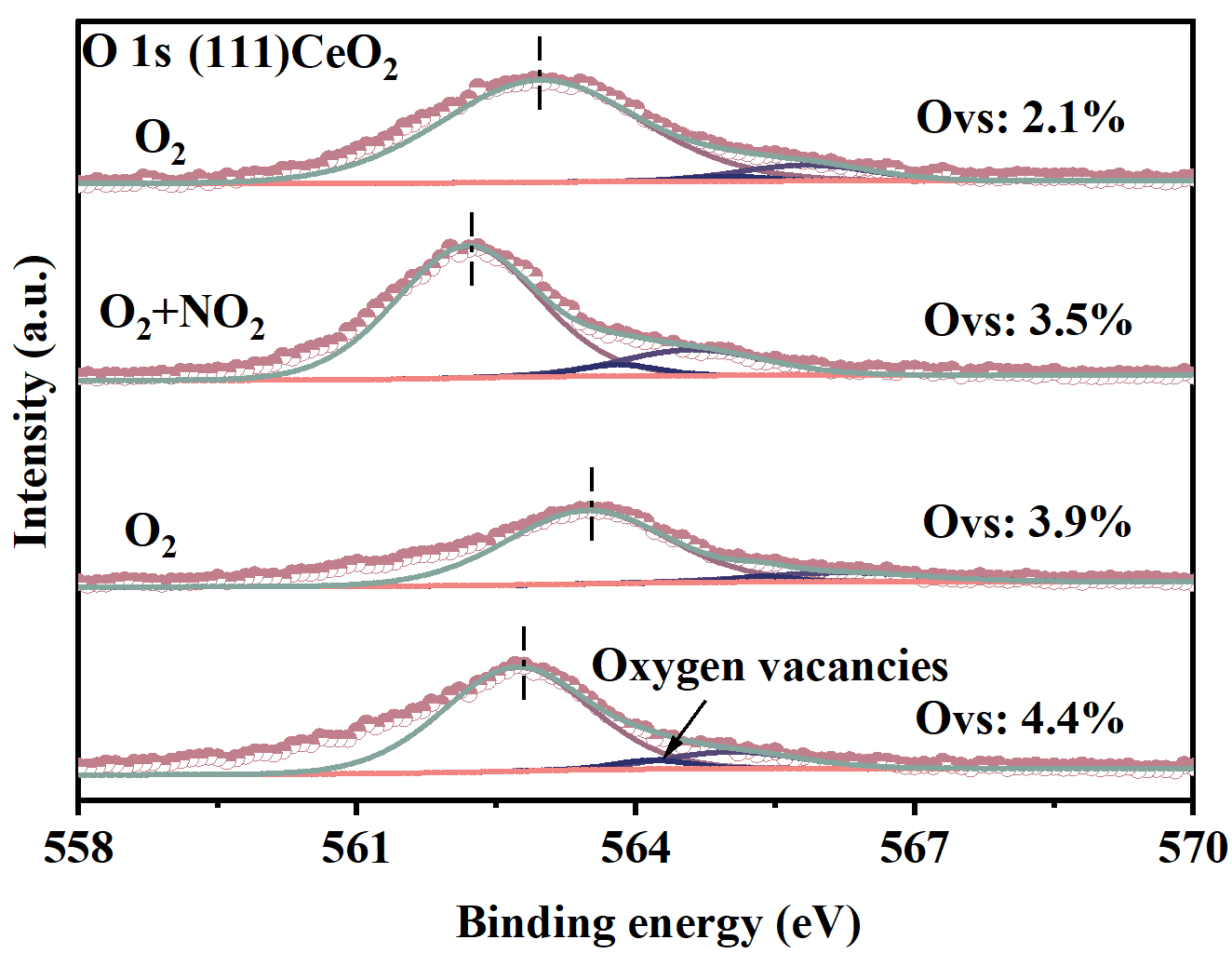


**Figure S34.** In situ NAP-XPS O 1s of (111)CeO_2_ recorded at room temperature after exposed to O_2_, NO_2_+O_2_ and O_2_.


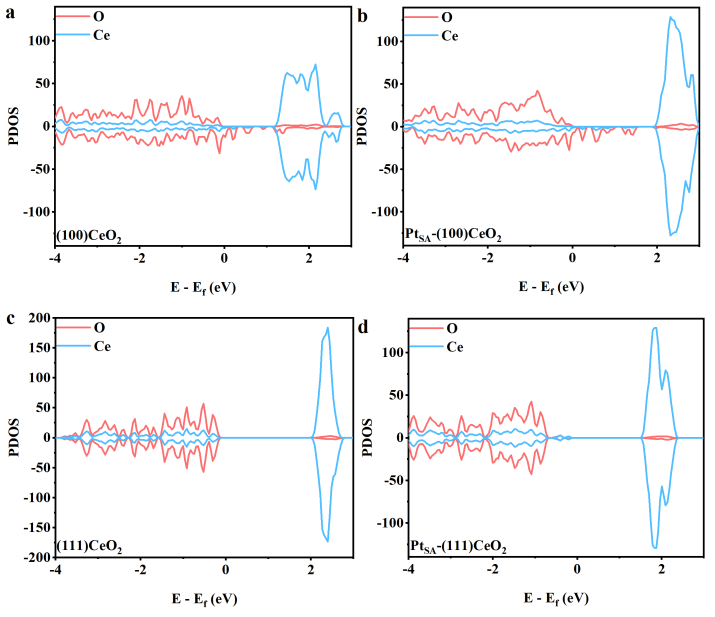


**Figure S35.** The PDOS of Ce and O before and after Pt adsorption. (a) (100)CeO_2_, (b) Pt_SA_-(100)CeO_2_, (c) (111)CeO_2_ and (d)Pt_SA_-(111)CeO_2_.


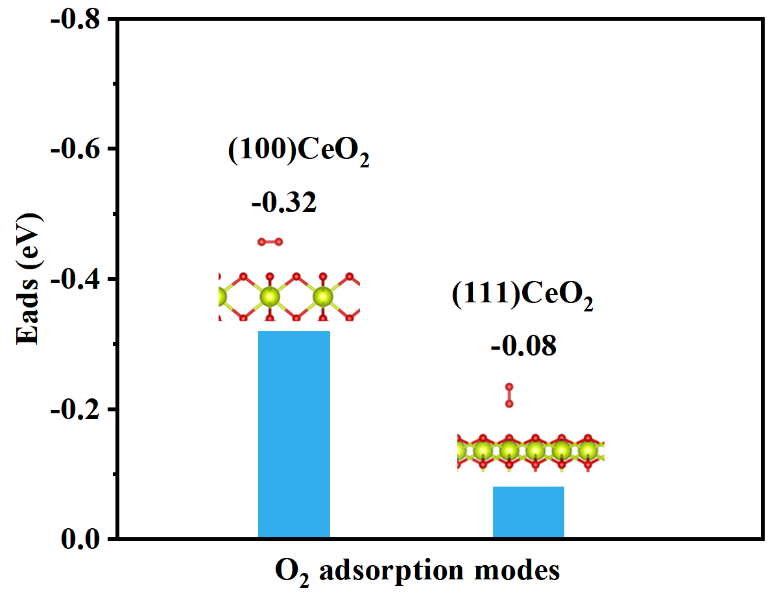


**Figure S36.** The adsorption energy of Pt_SA_-(100)CeO_2_ and Pt_SA_-(111)CeO_2_ toward O_2_.


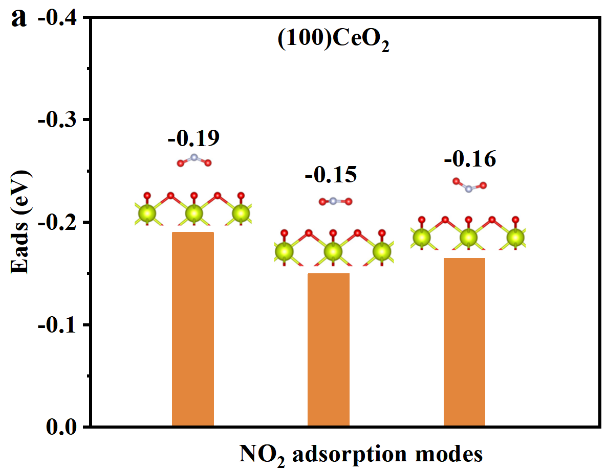

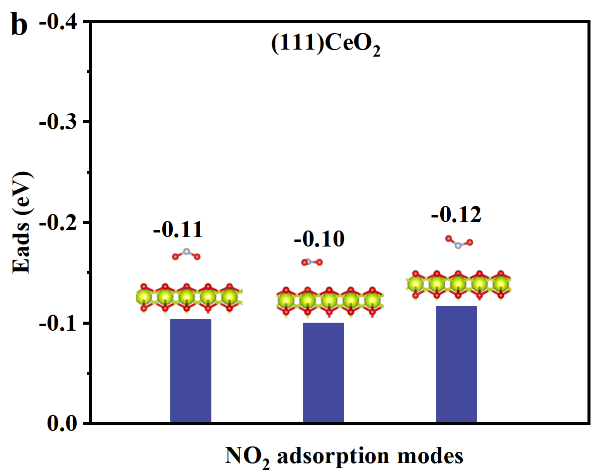


**Figure S37.** The adsorption energy of Pt_SA_-(100)CeO_2_ and Pt_SA_-(111)CeO_2_ toward NO_2_.


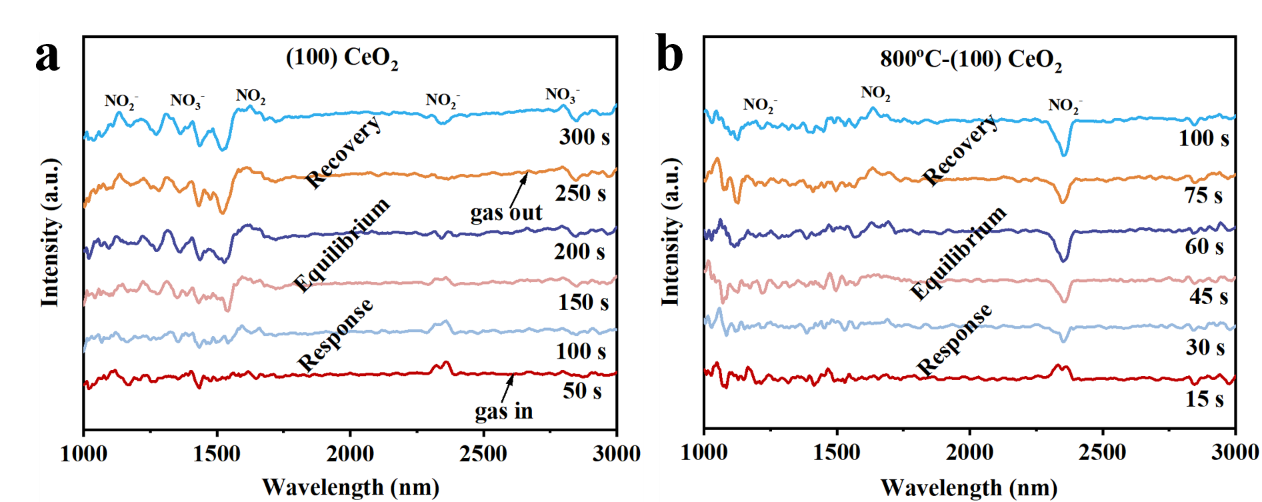


**Figure S38.** Gas sensing reaction on the surface of (100)CeO_2_ and 800^o^C-(100)CeO_2_.


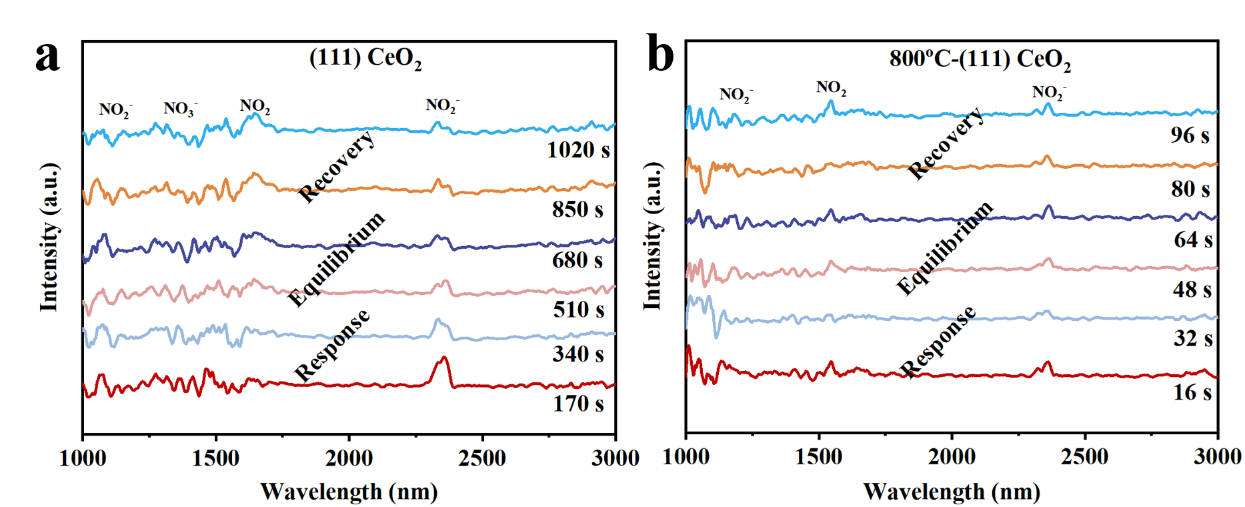


**Figure S39.** Gas sensing reaction on the surface of (111)CeO_2_ and 800^o^C-(111)CeO_2_.


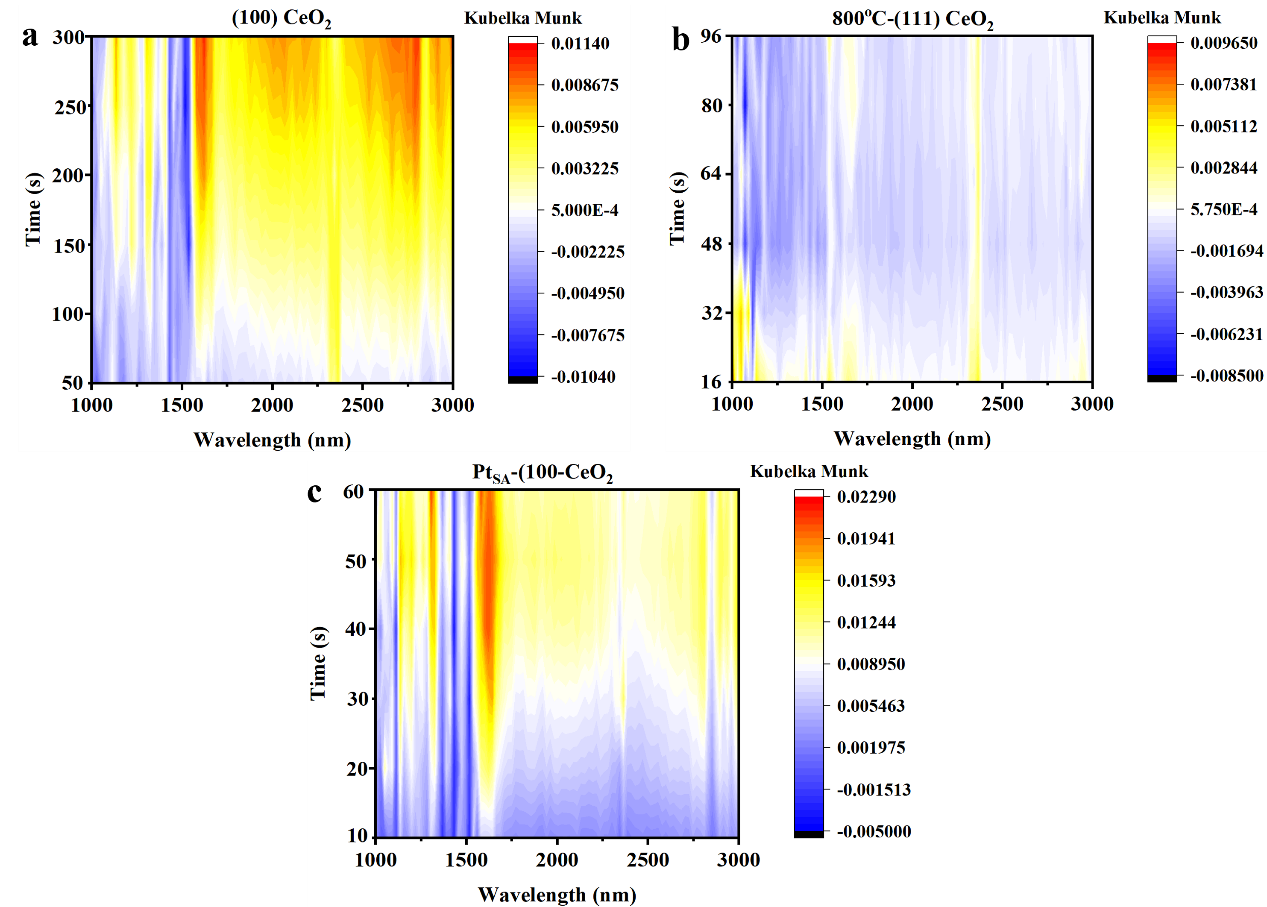


**Figure S40.** 2D pseudo-color In-situ FTIR spectra of (100)CeO_2_, 800^o^C-(100)CeO_2_ and Pt_SA_-(100)CeO_2_.


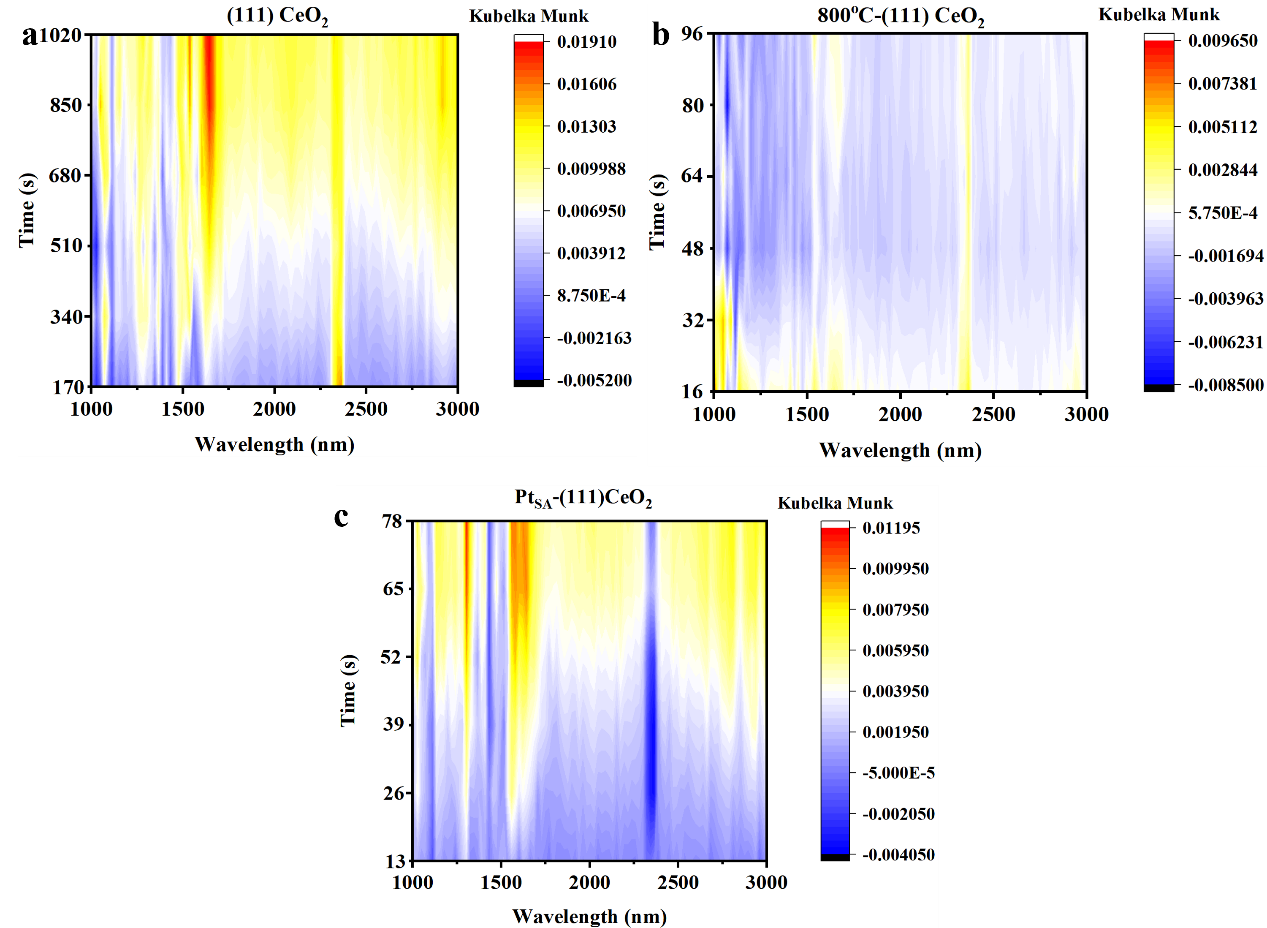


**Figure S41.** 2D pseudo-color In-situ FTIR spectra of (111)CeO_2_, 800^o^C-(111)CeO_2_ and Pt_SA_-(111)CeO_2_.
